# Synbiotic feed supplementation significantly improves lipid utilization and shows discrete effects on disease resistance in rainbow trout (*Oncorhynchus mykiss*)

**DOI:** 10.1038/s41598-020-73812-8

**Published:** 2020-10-12

**Authors:** Kasper Rømer Villumsen, Maki Ohtani, Torunn Forberg, Elisabeth Aasum, John Tinsley, Anders Miki Bojesen

**Affiliations:** 1 Preventive Veterinary Microbiology, Department of Veterinary and Animal Sciences, University of Copenhagen, Frederiksberg, Denmark; 2 BioMar Group, Trondheim, Norway; 3 Present Address: Division of Development of Functional Brain Activities,Research Centre for Child Mental Development, University of Fukui, Fukui, Japan

**Keywords:** Microbiology, Applied microbiology, Pathogens

## Abstract

Enteric redmouth disease caused by the bacterial pathogen *Yersinia ruckeri* is the main reason for antimicrobial prescription, and a cause of substantial economic losses and decreased animal welfare in aquaculture. Given the importance of the intestinal microbiota in digestion and disease, our aim was to investigate whether synbiotic feed supplementation strategies could improve feed performance and disease resistance. Four experimental synbiotic feeds formulated with pre- and probiotics were tested against a commercially available probiotic control feed. Each experimental feed was evaluated for feed performance, effects on gross as well as intestinal morphometrics, and finally their effect on resistance against a waterborne experimental infection with *Yersinia ruckeri* serotype O1, biotype 2. While co-supplementing *Pediococcus acidilactici* with citrus flavonoids or bacterial paraprobiotics significantly improved utilization of feed lipid content relative to the control group, a decrease in lipid utilization was observed for feeds that combined *P. acidilactici* with yeast paraprobiotics. No significant improvements on disease resistance were observed. Still, synbiotic formulations including *P. acidilactici* led to reduced risks relative to that of the control group, while an increased relative risk was observed for a *Bacillus*-based formulation. In conclusion, two of the synbiotic supplements significantly improved lipid utilization and contributed to minor increases in disease resistance.

## Introduction

As worldwide production of fish in aquaculture continues to grow, intense production schedules increase the risk of disease outbreaks. Enteric redmouth disease, caused by the bacterial pathogen *Yersinia ruckeri*^1,2^, currently accounts for more than 90% of the prescribed antimicrobials used in Danish aquaculture. In 2017, the total amount used was 1697 kg active substance^3^, for a total production of 48.300 tons^4^. Exceptionally high water temperatures presumeably led to increased outbreak severity and induced an increase of antimicrobial prescription of more than 100% from 2017 to 2018^3^. Prophylactic measures including vaccines^5–8^ and improved vaccination strategies^9,10^ have been developed and implemented. In spite of these efforts outbreaks continue to occur.

In an effort to improve feed performance and disease resistance, and thereby reduce the need for antimicrobial treatments and improve both animal welfare and farm revenue, feed supplements promoting disease resistance may add positively to existing prophylaxis and treatments strategies. In line with this concept, the aim of the present study was to test if feed performance and resistance towards infection with *Y. ruckeri* could be improved through synbiotic feed supplements.

Pre- pro- and synbiotic feed additives all have the same overall aim: the establishment of an intestinal microbiota that is beneficial to the health of the host. This is considered to occur through three modes of action. While prebiotics have recently been redefined as “substrates that are selectively utilized by host microorganisms conferring a health benefit”^11^, probiotics are defined as “live microorganisms, which when consumed in adequate amounts, confer a health effect on the host”^12,13^. Synbiotic feed supplementation is the co-supplementation of pre- and probiotics that benefits host health or welfare by selectively stimulating the growth of implanted, as well as other beneficial bacteria^14^. A separate and more recent derivative from the probiotics, paraprobiotics are defined as inactivated microbial cells or fractions thereof^15,16^. While microbial by nature, these do not match the probiotic definition. Throughout this study, paraprobiotics are considered to belong to the prebiotic category of feed supplements. They will, however, be referenced to and discussed as paraprobiotics.

Prebiotic supplementation of fish feed has proven beneficial for various compounds and for several species of fish. This includes postitive effects on different feed performance parameters following supplementation with a variety of compounds, including yeast cell wall components. Examples include yeast cell wall supplementation of Japanese seabass (*Lateolabrax japonicus*)^17^, fructose- and mannose-oligosaccharide (MOS) supplementation in Atlantic salmon (*Salmo salar*)^18^, as well as both MOS supplementation^19–21^ and incorporation of various organic acids in rainbow trout feeds either alone^22–25^, or in combination with β-glucans^26^. In addition to this, improved disease resistance towards bacterial infection has been reported for yeast cell wall supplements in Japanese seabass^17^, as well as citrus flavonoid, β-glucan, co-supplementation of β-glucan and organic acids in rainbow trout^26–28^.

Several probiotics have also proved beneficial in fish feeds. Proven effects include inhibitory activity against *Aeromonas hydropila*, *A. salmonicida*, *Vibrio anguillarum* and *Y. ruckeri* as demonstrated in vitro for *Lactococcus lactis*^29^, as well as reduced mortality in rainbow trout fed *Lactobacillus plantarum* supplements following *Lactococcus garvieae* infection. Studies performed using rainbow trout supplemented with *Pediococcus acidilactici* suggest that this probiotic is capable of colonizing the rainbow trout gastrointestinal (GI) tract^30,31^ and that it induces an increase in culturable, autochthonous bacterial counts from the GI tract, as well^32^. Paraprobiotics, potentially offering advantages found with probiotics, without the same regulatory restrictions on their use, have seen increasing intention in recent years^16^. Previous studies on grouper (*Epinephelus coioides*) and rainbow trout have demonstrated positive effects on feed performance following feed supplementation with inactivated *Bacillus pumilus* and *Enerococcus faecalis*, respectively^33,34^. In addition, in vitro studies have indicated an ability of yeast paraprobiotics to adsorb pathogenic bacteria, potentially inducing a beneficial effect on disease resistance^35^.

Previous investigations on combined synbiotic feed supplements in fish have been promising. When co-supplemented as a synbiotic mixture, *P. acidilactici* and galacto-oligosaccharides resulted in significantly increased final weights, weight gains, daily growth rates and feed conversion ratios in rainbow trout, while no significant differences were observed for neither the pre- nor the probiotic component when these were administered alone^32^. Synbiotic supplementation of *P. acidilactici* and MOS also had a positive, albeit not statistically significant, effect on the relative risk during infection with *Y. ruckeri* compared to control fed fish^36^.

To investigate the potential synergies of combining proven pre- and probiotics further, four experimental, proprietary synbiotic feed formulations were evaluated in the present study. Three were formulated with *P. acidilactici* as the probiotic component, as well as either citrus flavonoids, bacterial paraprobiotics or yeast paraprobiotics. The fourth feed was formulated with a novel *Bacillus spp.* probiotic candidate and yeast paraprobiotics. In order to assess the effects of the synbiotic strategy, the control group was fed a commercial feed supplemented with probiotic *P. acidilactici*. The experimental setup consisted of a 63-day experimental feeding period, of which the initial 34 days were feeding only, and the final 29 days included an experimental, waterborne infection with *Y. ruckeri* serotype O1, biotype 2^37^. Throughout the experimental period, feed performance and morphometrics were monitored.

## Materials and methods

### Fish

Rainbow trout eggs (AquaSearch FRESH strain, all-female, AquaSearch OVA, Billund, Denmark) were hatched and reared at the Bornholm Salmon Hatchery (Nexø, Denmark). The hatchery has a disease-free record and upon arrival, the eggs were disinfected using Desamar K30. Prior to experimental feeding, the fish were transported to the BioMar A/S research facilities (Hirtshals, Denmark), where the fish were sorted into 30 fiberglass tanks (150 L) in a fully recirculated system with biological filtering and physical filtering mechanisms, including a UV-filter. Subsequently, an 8-day acclimatization period was allowed. Water quality parameters were recorded daily throughout the experimental period (Table 1).

**Table 1:** Physical and chemical water quality parameters.

	NH_4_^+^ (mg/L)	NO_2_ (mg/L)	NO_3_ (mg/L)	pH	Temp. (°C)
Mean	0.55	0.18	18.09	7.69	14.09
Standard deviation ( ±)	0.69	0.44	11.78	0.44	1.29

### Experimental feeding and setup

Following the acclimatization period, experimental feeding was initiated. Five different feed groups were established. Each type of feed is described in Table 2. Five 150 L tanks holding 64–68 individuals (mean weight with standard deviation = 2.06 ± 0.07 g) were included per feed group, of which four were subjected to experimental infection, and one was kept as a non-infected control. During the experimental phase, all feed group identities were blinded.

**Table 2:** Experimental feed content for both pellet sizes.

	Control		PECF		PEBP		PEYP		BAYP	
Probiotic	*P. acidilactici*		*P. acidilactici*		*P. acidilactici*		*P. acidilactici*		*Bacillus* candidate	
Prebiotic	-		Citrus flavonoids 0.15%		Bacterial paraprobiotic 0.003%		0.15% yeast paraprobiotic		0.15% yeast paraprobiotic	
Pellet size (mm)	1.1	1.5	1.1	1.5	1.1	1.5	1.1	1.5	1.1	1.5
Protein (%)	49.6	53.8	51.5	53.5	49.9	54.7	49.7	54.6	48.8	54.7
Lipid/fat (%)	17.3	20.2	15	20.4	16.2	20.13	18	19.8	19.1	20.2

The total feeding period was 63 days, of which the experimental infection ran for the final 29 days. Daily feeding was 3.7% of total biomass up until the experimental infection, and 1.7% during the infection period. This decrease in feed percentage was made to accommodate a decrease in appetite during the infection, and to avoid the strain of excessive waste feed on the tank and filter setup. To accommodate increasing fish size, the fish were fed 1.1 mm pellets for the first 7 days of the feeding period, while 1.5 mm pellets were fed for the remainder of the experiment. Feed lipid and protein content was measured using near-infrared spectroscopy.

### Feed performance

Through days 0–34 of the feeding period leading up to the experimental infection, feed performance parameters were tracked for all five tanks in each feed group. Based on initial and endpoint bulk weight, counts of fish per tank, consumed feed and feed composition, the following parameters were calculated^38,39^:

1. Relative growth rate (RGR):
  $$RGR=\frac{(endpoint biomass (g)-initial biomass (g))}{initial biomass (g)} \times 100$$
2. Economic feed conversion ratio (eFCR):
  $$eFCR= \frac{consumed food (g)}{\left(endpoint biomass \left(g\right)-initial biomass(g)\right)+lost biomass (g)}$$
3. Specific growth rate (SGR):
  $$SGR=\left( \frac{\mathrm{ln}\left(\mathit{endpoint} \mathit{biomass} (g)\right)-\mathrm{ln}\left(initial biomass (g)\right)}{days fed}\right)\times 100$$
4. Lipid efficiency ratio (LER):
  $$LER= \frac{(endpoint biomass (g)-initial biomass (g))}{lipid/fat intake (g)}$$
5. Protein efficiency ratio (PER):
  $$PER= \frac{(endpoint biomass (g)-initial biomass (g))}{protein intake (g)}$$

### Sampling

All sampling was performed from tanks designated as non-infected controls to avoid interference with the experimental infection. Weight and fork length was recorded for ten convenience netted fish from each feed group at three different time points: day 0, day 34, and day 63. These time points correspond to immediately prior to feeding, immediately prior to start of the experimental infection in the infection tanks and the end of the experiment, respectively. In addition to weight and length measurements, ten individuals were convenience netted per group for sampling of intestinal tissue at day 0 and day 63. Immediately prior to sampling, each individual was euthanized in an overdose of benzocaine (Aquacen, Cenavisa, Spain). The gastrointestinal tract was subsequently exposed by a ventral midline incision. Adhering to the gross morphological description of the Atlantic salmon gastrointestinal tract^40^, tissue samples for histological examination were obtained from two distinct sections: A) spanning the distal section of the second mid-intestinal and posterior segments, and B) from the distal section of the first segment of the mid-intestine (see Supplementary Fig. S1). Following sampling, the tissues were immediately placed in 10% neutral buffered formalin for 24 h, and then changed to 70% ethanol for further storage.

### Morphometrics

At each of the three time points mentioned in the previous section, weight and length were utilized to calculate Fulton’s condition factor (*K*)^41,42^ for each individual:

6. Fulton’s condition factor (*K*):
  $$K= \frac{weight (g)}{{length (cm)}^{3}}\times 100$$

For each sampling time point, a relative condition factor was calculated. All individual condition factors were divided by the control group mean value at each time point.

Intestinal samples taken from the GI tract were processed for histological sectioning at the pathology lab of the Division of Diagnostics & Scientific Advice, Danish Technical University, Denmark. Following paraffin embedding, sections were cut and the tissues were stained using hematoxylin and eosin (H&E). Micrographs of one full H&E stained section of the posterior segment of the GI tract from each of the ten fish from the BAYP and PECF feed groups were captured using a Zeiss Axioplan2 microscope (Carl Zeiss Microscopy GmbH, Jena, Germany) fitted with a Zeiss Axiocam 702 mono (Carl Zeiss Microscopy GmbH, Jena, Germany) camera controlled by Zen 2 (Blue edition, Carl Zeiss Microscopy GmbH, Göttingen, Germany) software. Intestinal fold height was recorded from base to tip for all non-complex folds to avoid measuring posterior annulo-spiral valve heights, as described by Escaffre, et al*.*^43^. These measurements were performed using the segmented line tool in ImageJ (version 2.0.0-rc-68/1.52 h), following calibration of measurement tool using a calibration slide (0.01 mm, OMAX Microscope).

### Experimental *Y. ruckeri* infection

Bath infection was performed as previously described^37^. Briefly, cryopreserved *Y. ruckeri* serotype O1, biotype (strain 07111224) were streaked onto 5% blood agar plates and incubated at room temperature for 48 h. Subsequently Luria–Bertani media were inoculated with single colonies, and incubated for 36 h at room temperature with mechanical shaking or magnetic stirring. Prior to infection, all cultures were pelleted by centrifugation (3488 G, 15 min), the growth medium supernatant was discarded, and the bacteria were resuspended in tank water to 1:10 of their initial volume.

Fish in all infection tanks were anaesthetized and moved to designated infection containers holding 9 l of tank water. The infection was started by addition of 1 l of the 10X concentrated bacterial suspension to the respective infection containers to a final dose of 9.15 × 10^8^ colony forming units (CFU)/ml, as determined by plating of serially diluted infection suspension onto 5% blood agar plates and counting the colonies, accounting for dilutions. Following the 3-h infection period, all fish were netted and returned to their respective holding tanks and closely monitored several times per day in accordance with the experimental animal license for 29 days. Upon reaching the humane endpoints specified by the experimental animal license, fish were netted and euthanized in an overdose of benzocaine. The dorsal surface of the fish was sterilized in ethanol, and an incision through to the anterior kidney was made using a sterile scalpel and a smear from the anterior kidney onto 5% blood agar was made using a sterile inoculation loop. Resulting colonies were tested using a Bionor mono-aqua *Y. ruckeri* agglutination kit (Bionor AS, Skien, Norway) to verify that the pathogen could be re-isolated, satisfying Koch’s postulate. In this case, the individual was entered as an “event” in subsequent analyses, otherwise the individual would be censored from the time it left the experimental setup. Experimental feeding continued throughout the experimental infection period.

### Statistical analyses

All statistical analyses were performed using R (version 3.5.2 – “Eggshell Igloo”)^44^ run via RStudio (version 1.1.463). The following packages were used: ggplot2^45^, ggradar^46^, ggpubr^47^, survminer^48^, tibble^49^, RColorBrewer^50^, survival^51^, scales^52^, readxl^53^, fmsb^54^ and dplyr^55^.

A markdown.html document is available as supplementary material for full description of all analyses.

All statistical analyses were performed to either confirm or reject the null-hypothesis, that the data sets in question are equal, using an alpha-level of 0.05 as threshold.

All feed performance parameters including fish weight, length and Fulton’s condition factor data were checked for underlying Gaussian distribution of data through a Shapiro-Wilks test, as well as by visual inspection of QQ-plots (see supplementary markdown.html). Once this distribution was confirmed, data were analyzed by one-way ANOVA. If the result of the ANOVA was P < 0.05, multiple comparisons were performed using a Tukey post hoc test (95% family-wise conficence level). The performance data were summarized in radar chart and table form.

Intestinal fold heights were checked for underlying Gaussian distribution of data, as detailed for the performance parameters. As this distribution could not be proven, an unpaired Mann–Whitney U (non-parametric) test was used to compare feed groups at each time point.

Survival data were plotted using the Kaplan–Meier method. The survival curves for the quadruple replicate in each feed group were analyzed using the log-rank method, and as no statistically significant differences were demonstrated, the data for each feed code replicate was pooled. Given the ten possible comparisons, a Bonferroni-correction for multiple comparisons result in an alpha-level 0.05/10 = 0.005 for pairwise comparisons of survival curves.

Finally, the resulting survival curves for each feed code were compared using the log-rank method. Hazard-ratio analysis was then performed using the Cox proportional hazards method.

### Ethics Statement

The protocols involving use of experimental animals in this study were approved by the Danish Animal Experiments Council under license number 2015-15-0201-00645. The granted license is in accordance with the Danish law on animal experiments, as well as EU directive 2010/63.

## Results

### Feed performance data

All mean feed performance results following the initial 34 days of experimental feeding are shown in Table 3.

**Table 3:** Feed performance data for each experimental feed group. All values are group mean ± standard deviation. Statistically significant differences between feed groups following ANOVA are indicated by different lowercase letters. See “Materials and methods” section for details on statistical analyses.

	Control	PECF	PEBP	PEYP	BAYP
RGR	151.8 ± 4.3	149.6 ± 4.5	152.6 ± 4.0	149.9 ± 4.9	151.2 ± 5.3
eFCR	0.62 ± 0.02	0.62 ± 0.02	0.62 ± 0.02	0.63 ± 0.02	0.62 ± 0.02
SGR	2.73 ± 0.03	2.71 ± 0.03	2.74 ± 0.02	2.71 ± 0.02	2.72 ± 0.04
LER	9.33 ± 0.2^d^	10.80 ± 0.4^a^	10.0 ± 0.2^c^	8.87 ± 0.3^b,d^	8.43 ± 0.3^b^
PER	3.25 ± 0.1	3.14 ± 0.1	3.26 ± 0.1	3.21 ± 0.1	3.30 ± 0.1

**Figure 1:**
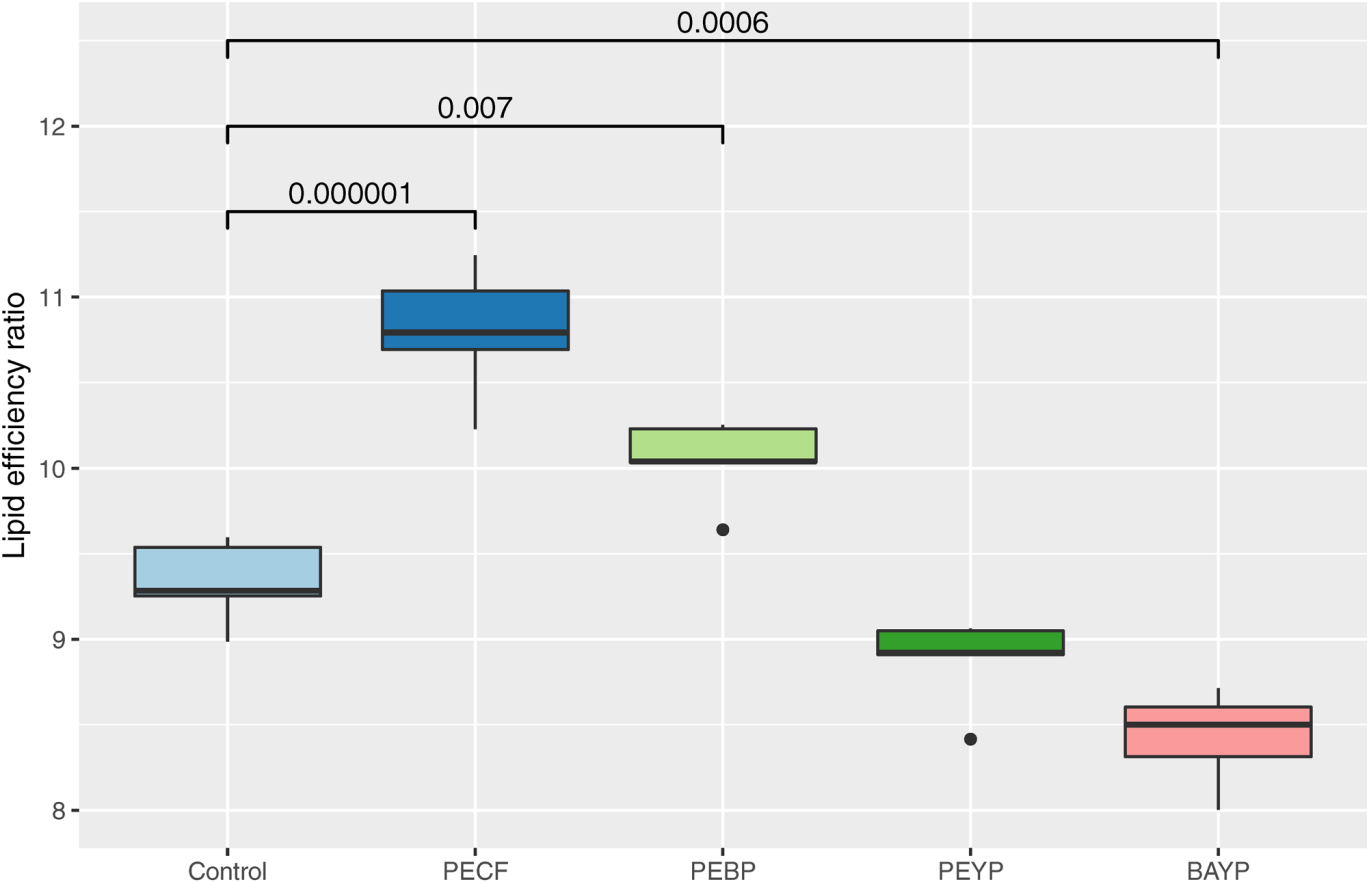
Lipid efficiency ratios for each group. The figure shows the median values with 25% and 75% quartiles (box) for all group replicates. Whiskers indicate largest and smallest observation equal to or less than 1.5 × interquartile range above and below the box, respectively. Outliers beyond these are shown individually. The *P* values shown are all based on comparisons with the control group, and are obtained by one-way ANOVA followed by Tukeys post hoc test for multiple comparisons (95% family-wise confidence level).

Across all calculated parameters, statistically significant differences were observed between group LERs. The PECF (P = 0.000001) and PEBP (P = 0.007) feed groups both displayed significantly elevated LERs relative to the control group (16% and 7%, respectively), while the PEYP was not significantly different from the control group. The BAYP group had a significantly lowered LER relative to the control (P = 0.0006). The LER values for each group are shown in Fig. 1.

### Morphometrics

**Figure 2:**
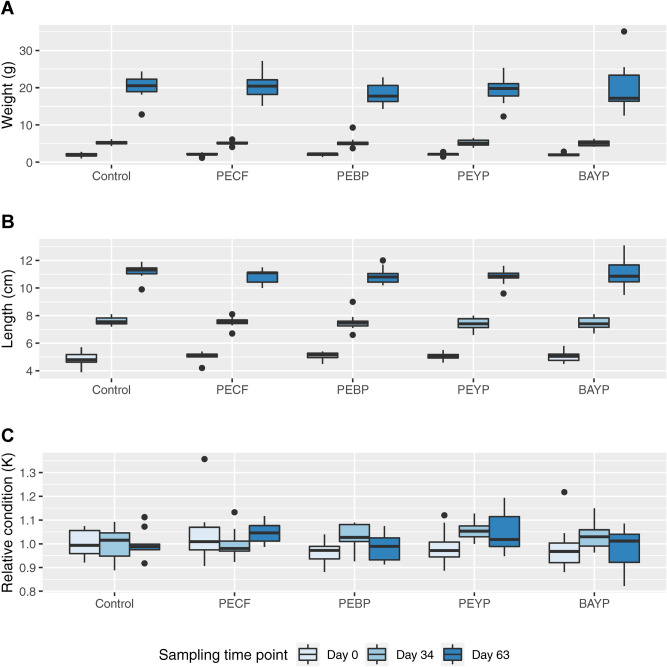
Gross morphometrics boxplot. Development of weight (**A**), length (**B**) and relative condition factor (**C**) throughout the experimental period. For details on sampling, measurements and calculation of K, see Materials & Methods. Each boxplot shows median value with 25% and 75% quartiles (box). Whiskers indicate largest and smallest observation equal to or less than 1.5 × interquartile range above and below the box, respectively. Outliers beyond these are shown individually.

Recorded gross measurements of weight and length, as well as their resulting condition factors are shown as boxplots in Fig. 2. Growth measured by either parameter was similar in all groups, or showed only very subtle differences, with no statistically significant differences identified between groups at any time point.

**Figure 3:**
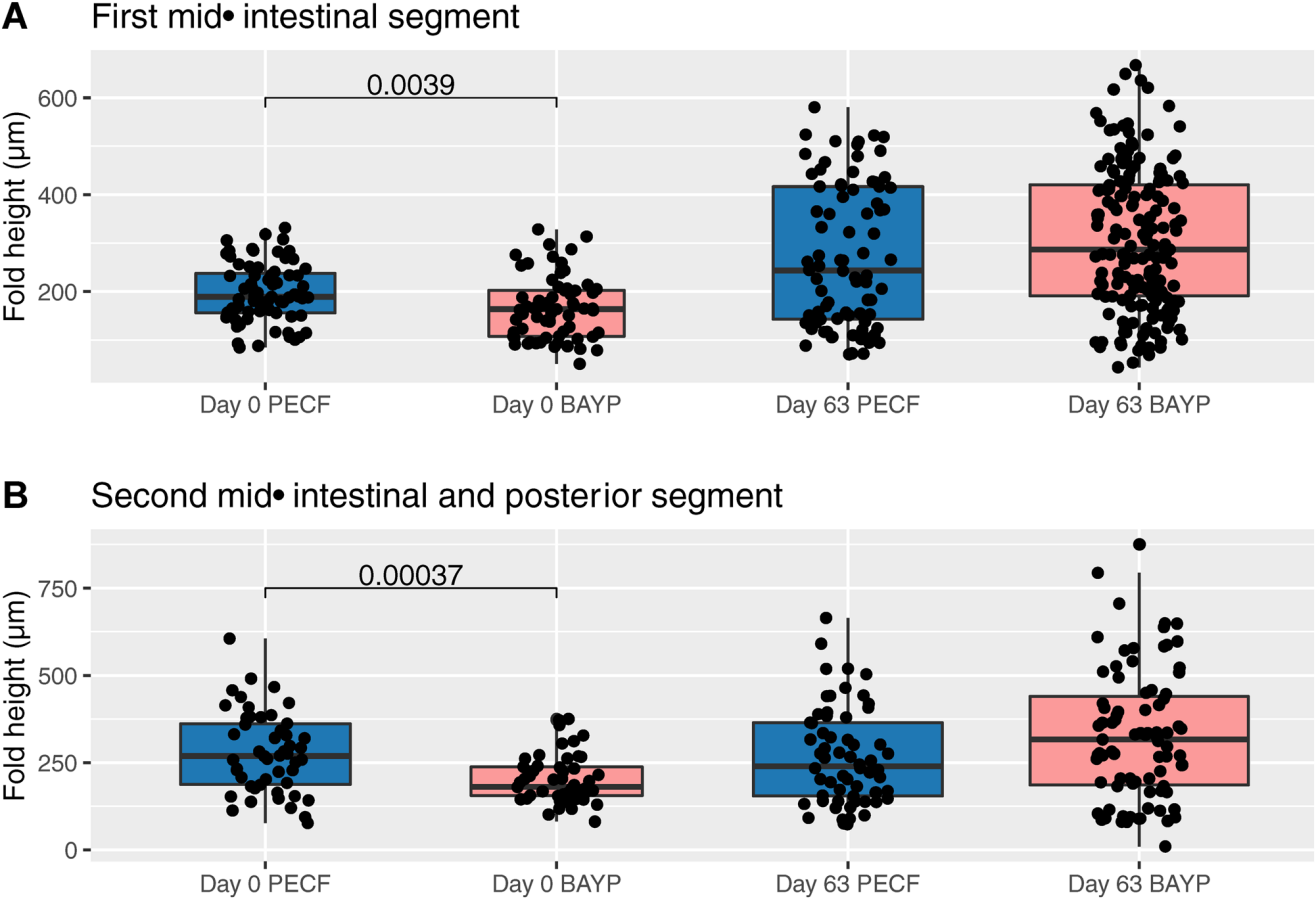
Intestinal morphometrics boxplot. Measured intestinal heights from (**A**) the first mid-intestinal segment, and (**B**) the second mid-intestinal and posterior segments immediately prior to the start of the feeding period, and at the end of the entire experimental period. For details on sampling and measurements, see Materials and Methods. Each boxplot shows median value with 25% and 75% quartiles (box). Whiskers indicate largest and smallest observation equal to or less than 1.5 × interquartile range above and below the box, respectively. Outliers beyond these are shown individually. Given that the numbers of individual measurements vary between groups, as well as time points, the width of each boxplot is increased with increasing numbers of measurements as an arbitrary measure to reflect these differences. Groups were compared for each segment at each sampling time point by Mann–Whitney U test. Statistically significant differences are indicated by *P* values.

Given the results from the lipid utilization, influences of feed supplements on intestinal morphology were assessed. The PECF and BAYP supplemented feed groups, representing the highest and lowest recorded LER, respectively, were selected for microscopical examination. The results are shown in Fig. 3. Comparing samples taken prior to experimental feeding and samples taken at the conclusion of the experimental feeding period, intestinal fold height was significantly higher in the PECF supplemented feed group, prior to any experimental feeding, for both the first and second mid-intestinal segments (P = 0.0039 and P = 0.00037, respectively). No differences were seen following the experimental feeding period.

### Experimental infection

**Figure 4:**
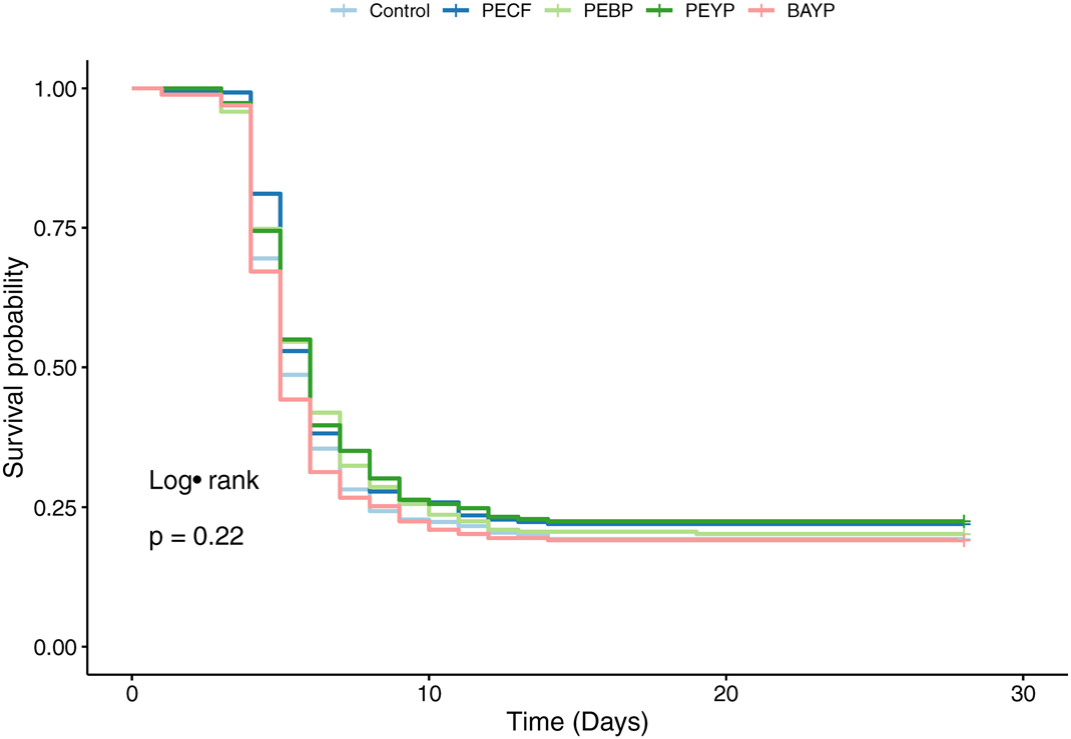
Survival curves from experimental infection. Kaplan–Meier plot of survival data showing computed survival probabilities for each feed group over time. All censored events are shown as ticks.

**Figure 5:**
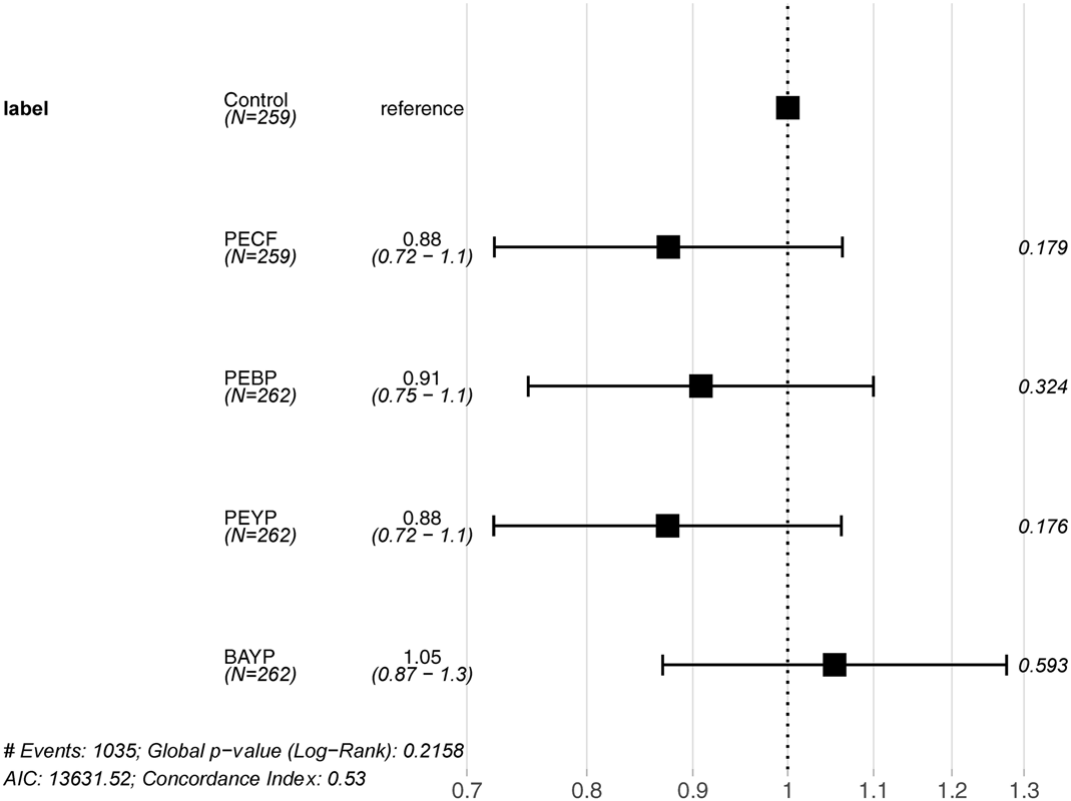
Forest plot of proportional hazards. Results from the Cox proportional hazards analysis. The control group has been set as reference. Each line shows the ratio of the hazard estimate of the given feed group relative to that of the control group ± 95% confidence interval, as well as the *P* value for that comparison.

A single individual was lost during the initial feeding phase, prior to the experimental infection. The survival curves obtained by pooling replicates following initial analyses reflect all mortalities observed during the experimental infection and are shown in Fig. 4. Endpoint mortalities for each group were: Control = 19.3%, PECF = 22%, PEBP = 20.2%, PEYP = 22.5% and BAYP = 19.1% (See supplementary Markdown.html). No significant differences were observed between any of the survival curves (P = 0.22), and no further pairwise comparisons were therefore warranted. A Cox proportional hazards analysis found no significant changes in relative risk for any of the synbiotic groups when compared to the probiotic control group (P > 0.1). However, all synbiotic formulations that include *P. acidilactici* showed a reduced risk level, whereas the BAYP group showed a slightly increased relative risk. While these ratios were not statistically significant, the pattern is clear from the results shown in Fig. 5.

## Discussion

With the aim of improving disease resistance and feed performance in rainbow trout, four experimental, synbiotic feed formulations were tested and compared to results obtained using a commercial probiotic feed. While individual combinations of pre- and probiotics featured individual strengths, general trends were also identified.

The main difference between feed groups in the present study, was observed with regards to feed performance. The primary outcome of the present study was that LER can be increased significantly (7–16%) through a combination of *P. acidilactici* as the probiotic component, and either citrus flavonoids or bacterial paraprobiotics as prebiotic components, as compared to inclusion of dietary *P. acidilactici* alone. This is a substantial and statistically significant improvement of feed utilization. Furthermore, given the general shift from marine- to plant-based sourcing of feed components^39^ and the potential effects of such a shift on fish muscle composition^56^, an increased efficiency in conversion of lipid to biomass must be considered highly beneficial in terms of efficient utilization of feed components and product quality. In a previous study, Baba et al*.* found a statistically significant increase in serum protein concentration, as well as significant reductions in serum triglyceride and cholesterol concentrations in Mozambique tilapia (*Oreochromis mossambicus*) fed crude citrus essential oil extracts^57^. Weight gain, SGR and FCR were, however, unaffected by the supplement. While the outer peel of citrus fruits have been shown to generally contain the highest concentrations of flavonoids, citrus peel essential oils remain a crude mix of many components^57,58^. In a recent study, prebiotic supplementation with citrus flavonoids alone had no apparent effects on weight gain, FCR, SGR, LER or PER in rainbow trout^26^. A plausible explanation for the improved LER observed in this study, could be the additional inclusion of probiotics, providing a synergistic improvement of lipid utilization.

A study on *E. faecalis* paraprobiotics has previously demonstrated improved final weight, weight gain and specific growth rate in rainbow trout^34^. While these were not found to be significantly elevated in the present study, the improved LER observed here likely reflects a similar beneficial effect of a bacterial paraprobiotic supplement on feed performance. It must be noted, that as with the abovementioned citrus oil extracts, paraprobiotic preparations should be considered crude in nature, and detailed information on their exact composition can be sparse, potentially resulting in variation between products and suppliers, as recently noted for yeast derived products^59^.

When comparing the probiotic control and the synbiotic alternatives, the results from the present study strongly indicate synergistic effects from the PECF and PEBP formulations. This is evident when comparing the lipid utilization of these two groups to that of the control and the PEYP groups. While supplementation with MOS, a major yeast cell wall component, has proven beneficial in previous performance studies in Atlantic salmon and rainbow trout^18–20,60^, the PEYP group in the present study was shown to have an LER similar to that of the control group. Potential antagonistic effects of the BAYP composition, however, cannot be dissected here due to the lack of a *Bacillus*-only group, but the significantly decreased LER observed in this group further underlines the effects of the composition of synbiotic feed supplements.

Whether the observed differences in LER affects total body composition in terms of fat percentage or -composition, was not investigated in the present study, but should be investigated in future studies.

Previous studies have reported intestinal morphological changes following probiotic^30,61^, as well as MOS feed supplementation^20,62^. While all experimental feeds in the present study are formulated with a probiotic supplement, differences in the prebiotic component of the synbiotic formulation could potentially affect the intestinal morphology. To examine this, a histological comparison of the intestinal fold heights for PECF and BAYP groups were performed, as these represented the highest and lowest LER recorded in the present study, respectively. A positive correlation has previously been demonstrated between fold height and epithelial surface in rainbow trout^43^. Differences in fold height could therefore indicate differences in total surface area within the intestinal sections examined. Interestingly, the only statistically significant differences found between the two groups were prior to experimental feeding, at day 0. All fish used in this setup were obtained from the same source and were fed the same feed at day 0. Furthermore, as shown in Fig. 2 there were no observed differences in length, weight or relative condition factor at day 0. The observed significant difference in intestinal fold height was unexpected, but could potentially be a result of the scattered nature of the observations, although P-values (< 0.01) indicate a low probability of the null hypothesis to be true. At day 63, following the experimental feeding period, no statistically significant differences were observed. While no micromorphological measurements were made in the present study, a plausible explanation for the observed increases in LER relative to the probiotic control feed group is that the synbiotic formulations of the PEBP and PECF promote a more beneficial metabolic profile for the intestinal microbiota^32^.

With regards to disease resistance, no differences were observed between groups for the survival curves from the experimental infection study. From the Cox proportional hazards analysis, however, a trend was observed, as all *P. acidilactici*-based synbiotic formulations conferred a decrease in relative risk, when compared to the control group. While not statistically significant, this trend was clear and consistent. As the probiotic control group feed was also formulated with *P. acidilactici*, this trend is attributed to the additional prebiotic components in these synbiotic formulations. No difference was found between the PEBP and PEYP groups, and as such whether the paraprobiotics were yeast or bacterial in nature did not seem to play a role. No mechanistic investigations were performed regarding the specifics of any inhibitory function of the included paraprobiotics on *Y. ruckeri*. However, as the binding between yeast paraprobiotics and various bacterial pathogens has been suggested to be strain-specific^35^, further studies are warranted to address whether or not improvements could be made with various bacterial paraprobiotics.

While no differences, significant or other, in hazard ratios were found for the PECF, PEBP and PEYP, a change of probiotic component did not yield the same results, as a noticeable difference was seen in the hazard ratio analysis for the PEYP and BAYP feed groups. The exact nature of the *Bacillus* probiotic used in this study is proprietary information. In a previous study, however, Ramos et al*.* tested a commercial probiotic supplement based on *Bacillus subtilis* and *B. cereus toyoi* in rainbow trout and found the combination to be “harmful”^61^.

Citrus flavonoids have been suggested to conduct an antimicrobial activity against Gram-negative bacteria^63^, as well as suppression of bacterial mechanisms involved in infection^64^. Furthermore, a previous study has shown that citrus flavonoid supplemented feed conferred a significant reduction in relative risk in trout following a similar waterborne infection with *Y. ruckeri*^26^. Rodriguez-Estrada et al*.* have previously demonstrated a seemingly dose-dependent reduction in mortality in rainbow trout fed MOS, inactivated *E. faecalis* or a combination of both following intraperitoneal injection of *A. salmonicida*^34^. While the statistical significance of these previous results did not appear to carry over entirely into the synbiotic formulations investigated in the present study, the observed apparent decrease in risk of the PECF, PEBP and PEYP feed groups compared to the probiotic control group, could argueably be due to a synergistic protective effect of the combined feed components.

In conclusion, a substantial, statistically significantly increase in feed lipid utilization was demonstrated for synbiotic co-supplementation of *P. acidilactici* and either citrus flavonoids (P = 0.000001) or bacterial paraprobiotics (P = 0.007) relative to the probiotic control feed. Improving the LER by 7–16%, these feed formulations could make a noticeable difference throughout the production value chain. In addition to this significant feed performance improvement, a clear but non-significant tendency towards reduced risk during infection was observed for synbiotic feed supplements combining *P. acidilactici* and either citrus flavonoids, bacterial paraprobiotics or yeast paraprobiotics. This demonstrates that considerable effects on feed performance, as well as minor, non-significant effects on disease resistance can be obtained applying the PECF or PEBP synbiotic feed supplements in rainbow trout feed. Whether additional synergistic effects can be achieved by adding multiple prebiotic components, such as co-supplementing both citrus flavonoids and bacterial paraprobiotics or even further complementing with additional prebiotics needs to be addressed in further studies.

## Supplementary information

Supplementary Information.

## Data Availability

The datasets generated during and/or analysed during the current study are summarized in the present publication. Raw data can be made available from the corresponding author on reasonable request.
