# Publisher Correction: Synbiotic feed supplementation significantly improves lipid utilization and shows discrete effects on disease resistance in rainbow trout (*Oncorhynchus mykiss*)

**DOI:** 10.1038/s41598-021-88891-4

**Published:** 2021-04-28

**Authors:** Kasper Rømer Villumsen, Maki Ohtani, Torunn Forberg, Elisabeth Aasum, John Tinsley, Anders Miki Bojesen

**Affiliations:** 1 Preventive Veterinary Microbiology, Department of Veterinary and Animal Sciences, University of Copenhagen, Frederiksberg, Denmark; 2 BioMar Group, Trondheim, Norway; 3 Present Address: Division of Development of Functional Brain Activities, Research Centre for Child Mental Development, University of Fukui, Fukui, Japan

Correction to: *Scientific Reports* 10.1038/s41598-020-73812-8, published online 12 October 2020

A Supplementary Information file containing statistical analyses, which was included with the initial submission, was omitted from the original version of this Article.

The Supplementary Information file is now linked to this correction notice.

## Supplementary Information

Supplementary Information.

