# Supplementary material for: Synbiotic feed supplementation significantly improves lipid utilization and shows discrete effects on disease resistance in rainbow trout (Oncorhynchus mykiss)
Source: Sci Rep. 2020 Oct 12;10:16993. doi: 10.1038/s41598-020-73812-8 (PMC7550352; doi:10.1038/s41598-020-73812-8)
Supplement: Supplementary file 1 — Supplementary Information. [file 41598_2020_73812_MOESM1_ESM.docx]

**Supplementary material**

*Supplementary figure S1: Tissues sampled for intestinal morphology assessment. Showing the span of the intestine from the posterior segment immediately prior to the cloaca on the left and the pyloric cecae on the right, two sampling locations are highlighted. (A) a span over the distal section of the second mid-intestinal and posterior segments. (B) the distal section of the first segment of the mid-intestine. Full lines on the underlying material indicate length in centimeters.For details on sampling and subsequent analyses, see Materials & Methods.*
