# Supplementary material for: Publisher Correction: Synbiotic feed supplementation significantly improves lipid utilization and shows discrete effects on disease resistance in rainbow trout (Oncorhynchus mykiss)
Source: Sci Rep. 2021 Apr 28;11:9479. doi: 10.1038/s41598-021-88891-4 (PMC8081722; doi:10.1038/s41598-021-88891-4)
Supplement: Supplementary file 1 — Supplementary Information. [file 41598_2021_88891_MOESM1_ESM.html]

Supplementary Information File


# Supplementary Information File

This markdown document represents the complete analysis platform for the study:

**Synbiotic feed supplementation significantly improves lipid utilization and shows discrete effects on disease resistance in rainbow trout (Oncorhynchus mykiss)**  
*Kasper Rømer Villumsen, Maki Ohtani, Torunn Forberg, Elisabeth Aasum, John Tinsley & Anders Miki Bojesen*

This document includes all initial data analyses, as well as final results and figures.

# Table of contents

1. Loading packages
2. Feed performance analysis
3. Lipid efficiency ratio figure
4. Morphometrics
5. Survival analysis
6. Citations

## Loading packages:

The following packages were used in analyses performed in the present study.

```
library(ggplot2)
library(survival)
library(survminer)
library(readxl)
library(ggpubr)
library(dplyr)
library(RColorBrewer)
library(fmsb)
```

# Feed performance analysis

For each performance parameter, details regarding their calculations can be found in the materials and methods section of the published article. They were subsequently analyzed as follows:

- Summarizing statistics were calculated for each group based on data from each replicate tank in each group.
- Tankwise parameters for each group were assessed for Gaussian distibution of data (Shapiro-Wilk Normality test + visual inspection of QQ-plots).
- Statistical analyses were then performed based on the result.

## Relative growth rate (RGR):

Summarizing RGR data for each group:

### PECF:

```
##    Min. 1st Qu.  Median    Mean 3rd Qu.    Max. 
##   143.1   147.9   150.0   149.6   151.5   155.2
```

```
## 
##  Shapiro-Wilk normality test
## 
## data:  RGR$Blue
## W = 0.99048, p-value = 0.9813
```

### PEYP:

```
##    Min. 1st Qu.  Median    Mean 3rd Qu.    Max. 
##   141.4   150.8   151.1   149.9   153.0   153.1
```

```
## 
##  Shapiro-Wilk normality test
## 
## data:  RGR$Orange
## W = 0.73307, p-value = 0.02059
```

Visual inspection of the QQ-plot suggests that the result of the Shapiro-Wilk test is due to a single outlier.

### BAYP:

```
##    Min. 1st Qu.  Median    Mean 3rd Qu.    Max. 
##   143.1   148.9   152.6   151.2   154.8   156.3
```

```
## 
##  Shapiro-Wilk normality test
## 
## data:  RGR$Green
## W = 0.92927, p-value = 0.5914
```

### Control:

```
##    Min. 1st Qu.  Median    Mean 3rd Qu.    Max. 
##   145.6   150.4   151.3   151.8   155.7   156.1
```

```
## 
##  Shapiro-Wilk normality test
## 
## data:  RGR$Red
## W = 0.91794, p-value = 0.5167
```

### PEBP:

```
##    Min. 1st Qu.  Median    Mean 3rd Qu.    Max. 
##   146.2   152.2   153.0   152.6   155.8   156.0
```

```
## 
##  Shapiro-Wilk normality test
## 
## data:  RGR$Yellow
## W = 0.86146, p-value = 0.2335
```

Given the results from the tests for Gaussian distributions, a one-way ANOVA is deemed appropriate, as visual inspection of QQplots suggest that the one P-value <0.05 was due to a single outlier:

```
##             Df Sum Sq Mean Sq F value Pr(>F)
## tm           4   33.4   8.361   0.394   0.81
## Residuals   20  424.1  21.207
```

No significant differences are found during the ANOVA.

## Economic Feed Conversion Ratio (eFCR)

Summarizing eFCR data for each group:

### PECF:

```
##    Min. 1st Qu.  Median    Mean 3rd Qu.    Max. 
##  0.5944  0.6057  0.6193  0.6196  0.6251  0.6536
```

```
## 
##  Shapiro-Wilk normality test
## 
## data:  efcr$Blue
## W = 0.96213, p-value = 0.8227
```

### PEYP:

```
##    Min. 1st Qu.  Median    Mean 3rd Qu.    Max. 
##  0.6130  0.6140  0.6227  0.6267  0.6235  0.6601
```

```
## 
##  Shapiro-Wilk normality test
## 
## data:  efcr$Orange
## W = 0.75732, p-value = 0.03474
```

While the p-value is < 0.05, this is presumably a result of one outlier and low number of observations (see QQ-plot).

### BAYP:

```
##    Min. 1st Qu.  Median    Mean 3rd Qu.    Max. 
##  0.6005  0.6082  0.6156  0.6186  0.6296  0.6392
```

```
## 
##  Shapiro-Wilk normality test
## 
## data:  efcr$Green
## W = 0.96359, p-value = 0.8327
```

### Control:

```
##    Min. 1st Qu.  Median    Mean 3rd Qu.    Max. 
##  0.6024  0.6061  0.6227  0.6198  0.6246  0.6432
```

```
## 
##  Shapiro-Wilk normality test
## 
## data:  efcr$Red
## W = 0.93272, p-value = 0.6151
```

### PEBP:

```
##    Min. 1st Qu.  Median    Mean 3rd Qu.    Max. 
##  0.6020  0.6035  0.6148  0.6151  0.6151  0.6403
```

```
## 
##  Shapiro-Wilk normality test
## 
## data:  efcr$Yellow
## W = 0.8475, p-value = 0.1867
```

Visual inspections of QQ-plots and Shapiro-Wilk tests suggest Gaussian distributions, a one-way ANOVA is deemed appropriate.

```
##             Df   Sum Sq   Mean Sq F value Pr(>F)
## tm           4 0.000351 0.0000879    0.27  0.894
## Residuals   20 0.006504 0.0003252
```

No significant differences were found.

## Specific growth rate (SGR):

Summarizing SGR data for each group:

### PECF:

```
##    Min. 1st Qu.  Median    Mean 3rd Qu.    Max. 
##   2.670   2.692   2.695   2.705   2.713   2.756
```

```
## 
##  Shapiro-Wilk normality test
## 
## data:  SGR$Blue
## W = 0.92765, p-value = 0.5804
```

### PEYP:

```
##    Min. 1st Qu.  Median    Mean 3rd Qu.    Max. 
##   2.671   2.705   2.708   2.709   2.730   2.732
```

```
## 
##  Shapiro-Wilk normality test
## 
## data:  SGR$Orange
## W = 0.8895, p-value = 0.3546
```

### BAYP:

```
##    Min. 1st Qu.  Median    Mean 3rd Qu.    Max. 
##   2.682   2.692   2.726   2.724   2.751   2.768
```

```
## 
##  Shapiro-Wilk normality test
## 
## data:  SGR$Green
## W = 0.93293, p-value = 0.6165
```

### Control:

```
##    Min. 1st Qu.  Median    Mean 3rd Qu.    Max. 
##   2.700   2.710   2.723   2.732   2.761   2.766
```

```
## 
##  Shapiro-Wilk normality test
## 
## data:  SGR$Red
## W = 0.88091, p-value = 0.3135
```

### PEBP:

```
##    Min. 1st Qu.  Median    Mean 3rd Qu.    Max. 
##   2.721   2.730   2.730   2.742   2.763   2.765
```

```
## 
##  Shapiro-Wilk normality test
## 
## data:  SGR$Yellow
## W = 0.8205, p-value = 0.1178
```

Given the results from the tests for Gaussian distributions, a one-way ANOVA is deemed appropriate:

```
##             Df   Sum Sq   Mean Sq F value Pr(>F)
## tm           4 0.004694 0.0011734   1.356  0.284
## Residuals   20 0.017313 0.0008656
```

No significant differences were found during the ANOVA.

## Lipid efficiency ratio (LER):

Summarizing LER data for each group:

### PECF:

```
##    Min. 1st Qu.  Median    Mean 3rd Qu.    Max. 
##   10.23   10.69   10.79   10.80   11.04   11.24
```

```
## 
##  Shapiro-Wilk normality test
## 
## data:  LER$Blue
## W = 0.97157, p-value = 0.8852
```

### PEYP:

```
##    Min. 1st Qu.  Median    Mean 3rd Qu.    Max. 
##   8.416   8.910   8.921   8.872   9.048   9.063
```

```
## 
##  Shapiro-Wilk normality test
## 
## data:  LER$Orange
## W = 0.76838, p-value = 0.04366
```

Following visual inspection, the result of the Shapiro-Wilk test is deemed to be due to a single outlier.

### BAYP:

```
##    Min. 1st Qu.  Median    Mean 3rd Qu.    Max. 
##   8.003   8.312   8.500   8.426   8.603   8.714
```

```
## 
##  Shapiro-Wilk normality test
## 
## data:  LER$Green
## W = 0.94496, p-value = 0.7012
```

### Control:

```
##    Min. 1st Qu.  Median    Mean 3rd Qu.    Max. 
##   8.987   9.254   9.283   9.331   9.537   9.596
```

```
## 
##  Shapiro-Wilk normality test
## 
## data:  LER$Red
## W = 0.93504, p-value = 0.6311
```

### PEBP:

```
##    Min. 1st Qu.  Median    Mean 3rd Qu.    Max. 
##   9.641  10.035  10.040  10.040  10.229  10.253
```

```
## 
##  Shapiro-Wilk normality test
## 
## data:  LER$Yellow
## W = 0.85593, p-value = 0.214
```

Given the results from the tests for Gaussian distributions, a one-way ANOVA is deemed appropriate, as visual inspection of QQ-plots suggest that the one P-value <0.05 was due to a single outlier.

ANOVA of LER:

```
##             Df Sum Sq Mean Sq F value   Pr(>F)    
## tm           4 17.768   4.442   53.38 2.15e-10 ***
## Residuals   20  1.664   0.083                     
## ---
## Signif. codes:  0 '***' 0.001 '**' 0.01 '*' 0.05 '.' 0.1 ' ' 1
```

```
##   Tukey multiple comparisons of means
##     95% family-wise confidence level
## 
## Fit: aov(formula = r ~ tm)
## 
## $tm
##                    diff         lwr        upr     p adj
## PEYP-PECF    -1.9270489 -2.47298941 -1.3811085 0.0000000
## BAYP-PECF    -2.3722538 -2.91819423 -1.8263133 0.0000000
## Control-PECF -1.4674430 -2.01338347 -0.9215025 0.0000010
## PEBP-PECF    -0.7588411 -1.30478159 -0.2129007 0.0039379
## BAYP-PEYP    -0.4452048 -0.99114528  0.1007357 0.1453264
## Control-PEYP  0.4596059 -0.08633452  1.0055464 0.1258772
## PEBP-PEYP     1.1682078  0.62226736  1.7141483 0.0000271
## Control-BAYP  0.9048108  0.35887029  1.4507512 0.0006460
## PEBP-BAYP     1.6134126  1.06747217  2.1593531 0.0000002
## PEBP-Control  0.7086019  0.16266142  1.2545423 0.0073062
```

Several statistically significant differences were found, as seen in the summary above.

## PER

Summarizing PER data for each group:

### PECF:

```
##    Min. 1st Qu.  Median    Mean 3rd Qu.    Max. 
##   2.971   3.106   3.135   3.137   3.206   3.266
```

```
## 
##  Shapiro-Wilk normality test
## 
## data:  PER$Blue
## W = 0.97157, p-value = 0.8852
```

### PEYP:

```
##    Min. 1st Qu.  Median    Mean 3rd Qu.    Max. 
##   3.048   3.227   3.231   3.213   3.277   3.282
```

```
## 
##  Shapiro-Wilk normality test
## 
## data:  PER$Orange
## W = 0.76838, p-value = 0.04366
```

Visual inspection of the QQ-plot suggests that the result of the Shapiro-Wilk test is due to a single outlier.

### PEYP:

```
##    Min. 1st Qu.  Median    Mean 3rd Qu.    Max. 
##   3.134   3.255   3.329   3.300   3.369   3.412
```

```
## 
##  Shapiro-Wilk normality test
## 
## data:  PER$Green
## W = 0.94496, p-value = 0.7012
```

### Control:

```
##    Min. 1st Qu.  Median    Mean 3rd Qu.    Max. 
##   3.134   3.228   3.238   3.255   3.326   3.347
```

```
## 
##  Shapiro-Wilk normality test
## 
## data:  PER$Red
## W = 0.93504, p-value = 0.6311
```

### PEBP:

```
##    Min. 1st Qu.  Median    Mean 3rd Qu.    Max. 
##   3.130   3.258   3.260   3.259   3.321   3.329
```

```
## 
##  Shapiro-Wilk normality test
## 
## data:  PER$Yellow
## W = 0.85593, p-value = 0.214
```

Given the results from the tests for Gaussian distributions, a one-way ANOVA is deemed appropriate, as visual inspection of QQplots suggest that the one P-value <0.05 was due to a single outlier:

```
##             Df  Sum Sq  Mean Sq F value Pr(>F)
## tm           4 0.07633 0.019082   2.019   0.13
## Residuals   20 0.18899 0.009449
```

No significant differences are found from the ANOVA.

# Lipid efficiency ratio figure:

Visualization of the significantly different LER values:

```
## # A tibble: 25 x 2
##    group    data
##    <chr>   <dbl>
##  1 Control  9.54
##  2 Control  9.28
##  3 Control  9.25
##  4 Control  9.60
##  5 Control  8.99
##  6 PECF    11.2 
##  7 PECF    10.7 
##  8 PECF    10.8 
##  9 PECF    11.0 
## 10 PECF    10.2 
## # … with 15 more rows
```

# Morphometrics

## Weight, length and Fultons condition factor:

Descriptive statistics for each group for three different time points: pre-feeding, pre-infection and post infection:

```
##    Min. 1st Qu.  Median    Mean 3rd Qu.    Max. 
##   1.170   2.058   2.115   2.091   2.308   2.630
```

```
## 
##  Shapiro-Wilk normality test
## 
## data:  pre_feed_metrics$blue_weight
## W = 0.89981, p-value = 0.218
```

```
##    Min. 1st Qu.  Median    Mean 3rd Qu.    Max. 
##    4.20    5.00    5.10    4.97    5.20    5.40
```

```
## 
##  Shapiro-Wilk normality test
## 
## data:  pre_feed_metrics$blue_length
## W = 0.77074, p-value = 0.006385
```

```
##    Min. 1st Qu.  Median    Mean 3rd Qu.    Max. 
##  0.9066  0.9742  1.0085  1.0373  1.0694  1.3566
```

```
## 
##  Shapiro-Wilk normality test
## 
## data:  pre_feed_metrics$blue_cond
## W = 0.80501, p-value = 0.01668
```

```
##    Min. 1st Qu.  Median    Mean 3rd Qu.    Max. 
##   3.890   4.615   5.140   5.210   5.900   6.450
```

```
## 
##  Shapiro-Wilk normality test
## 
## data:  pre_feed_metrics$orange_weight
## W = 0.93086, p-value = 0.4564
```

```
##    Min. 1st Qu.  Median    Mean 3rd Qu.    Max. 
##   4.600   4.925   5.050   5.040   5.175   5.500
```

```
## 
##  Shapiro-Wilk normality test
## 
## data:  pre_feed_metrics$orange_length
## W = 0.94361, p-value = 0.5939
```

```
##    Min. 1st Qu.  Median    Mean 3rd Qu.    Max. 
##  0.8871  0.9448  0.9719  0.9877  1.0071  1.1200
```

```
## 
##  Shapiro-Wilk normality test
## 
## data:  pre_feed_metrics$orange_cond
## W = 0.91348, p-value = 0.3057
```

```
##    Min. 1st Qu.  Median    Mean 3rd Qu.    Max. 
##   1.700   1.825   1.990   2.051   2.167   2.830
```

```
## 
##  Shapiro-Wilk normality test
## 
## data:  pre_feed_metrics$green_weight
## W = 0.86216, p-value = 0.08092
```

```
##    Min. 1st Qu.  Median    Mean 3rd Qu.    Max. 
##    4.50    4.75    5.05    5.04    5.20    5.80
```

```
## 
##  Shapiro-Wilk normality test
## 
## data:  pre_feed_metrics$green_length
## W = 0.95722, p-value = 0.7538
```

```
##    Min. 1st Qu.  Median    Mean 3rd Qu.    Max. 
##  0.8805  0.9208  0.9676  0.9828  1.0033  1.2173
```

```
## 
##  Shapiro-Wilk normality test
## 
## data:  pre_feed_metrics$green_cond
## W = 0.85934, p-value = 0.07494
```

```
##    Min. 1st Qu.  Median    Mean 3rd Qu.    Max. 
##   1.040   1.692   1.810   1.933   2.292   2.790
```

```
## 
##  Shapiro-Wilk normality test
## 
## data:  pre_feed_metrics$red_weight
## W = 0.97341, p-value = 0.9206
```

```
##    Min. 1st Qu.  Median    Mean 3rd Qu.    Max. 
##   3.900   4.625   4.800   4.880   5.175   5.700
```

```
## 
##  Shapiro-Wilk normality test
## 
## data:  pre_feed_metrics$red_length
## W = 0.97278, p-value = 0.9154
```

```
##    Min. 1st Qu.  Median    Mean 3rd Qu.    Max. 
##  0.9209  0.9587  0.9935  1.0000  1.0561  1.0745
```

```
## 
##  Shapiro-Wilk normality test
## 
## data:  pre_feed_metrics$red_cond
## W = 0.91303, p-value = 0.3025
```

```
##    Min. 1st Qu.  Median    Mean 3rd Qu.    Max. 
##   1.400   1.905   2.260   2.060   2.270   2.400
```

```
## 
##  Shapiro-Wilk normality test
## 
## data:  pre_feed_metrics$yellow_weight
## W = 0.80851, p-value = 0.01839
```

```
##    Min. 1st Qu.  Median    Mean 3rd Qu.    Max. 
##   4.500   4.950   5.150   5.060   5.275   5.400
```

```
## 
##  Shapiro-Wilk normality test
## 
## data:  pre_feed_metrics$yellow_length
## W = 0.87404, p-value = 0.1114
```

```
##    Min. 1st Qu.  Median    Mean 3rd Qu.    Max. 
##  0.8815  0.9365  0.9723  0.9644  0.9892  1.0395
```

```
## 
##  Shapiro-Wilk normality test
## 
## data:  pre_feed_metrics$yellow_cond
## W = 0.95383, p-value = 0.7139
```

```
##    Min. 1st Qu.  Median    Mean 3rd Qu.    Max. 
##   4.090   4.963   5.115   5.125   5.322   6.100
```

```
## 
##  Shapiro-Wilk normality test
## 
## data:  pre_inf_metrics$blue_weight
## W = 0.96387, p-value = 0.8289
```

```
##    Min. 1st Qu.  Median    Mean 3rd Qu.    Max. 
##   6.700   7.425   7.550   7.530   7.675   8.100
```

```
## 
##  Shapiro-Wilk normality test
## 
## data:  pre_inf_metrics$blue_length
## W = 0.92699, p-value = 0.419
```

```
##    Min. 1st Qu.  Median    Mean 3rd Qu.    Max. 
##  0.9232  0.9686  0.9800  0.9994  1.0113  1.1327
```

```
## 
##  Shapiro-Wilk normality test
## 
## data:  pre_inf_metrics$blue_cond
## W = 0.89128, p-value = 0.1753
```

```
##    Min. 1st Qu.  Median    Mean 3rd Qu.    Max. 
##   3.890   4.615   5.140   5.210   5.900   6.450
```

```
## 
##  Shapiro-Wilk normality test
## 
## data:  pre_inf_metrics$orange_weight
## W = 0.95988, p-value = 0.7845
```

```
##    Min. 1st Qu.  Median    Mean 3rd Qu.    Max. 
##   6.600   7.125   7.400   7.420   7.775   8.000
```

```
## 
##  Shapiro-Wilk normality test
## 
## data:  pre_inf_metrics$orange_length
## W = 0.9535, p-value = 0.7099
```

```
##    Min. 1st Qu.  Median    Mean 3rd Qu.    Max. 
##  0.9998  1.0298  1.0530  1.0550  1.0751  1.1270
```

```
## 
##  Shapiro-Wilk normality test
## 
## data:  pre_inf_metrics$orange_cond
## W = 0.97383, p-value = 0.9239
```

```
##    Min. 1st Qu.  Median    Mean 3rd Qu.    Max. 
##   4.150   4.425   5.100   5.134   5.647   6.270
```

```
## 
##  Shapiro-Wilk normality test
## 
## data:  pre_inf_metrics$green_weight
## W = 0.9242, p-value = 0.3933
```

```
##    Min. 1st Qu.  Median    Mean 3rd Qu.    Max. 
##   6.700   7.150   7.400   7.440   7.825   8.100
```

```
## 
##  Shapiro-Wilk normality test
## 
## data:  pre_inf_metrics$green_length
## W = 0.96038, p-value = 0.7902
```

```
##    Min. 1st Qu.  Median    Mean 3rd Qu.    Max. 
##  0.9630  0.9904  1.0298  1.0342  1.0590  1.1493
```

```
## 
##  Shapiro-Wilk normality test
## 
## data:  pre_inf_metrics$green_cond
## W = 0.94786, p-value = 0.6433
```

```
##    Min. 1st Qu.  Median    Mean 3rd Qu.    Max. 
##   4.350   4.930   5.205   5.273   5.513   6.170
```

```
## 
##  Shapiro-Wilk normality test
## 
## data:  pre_inf_metrics$red_weight
## W = 0.97062, p-value = 0.8966
```

```
##    Min. 1st Qu.  Median    Mean 3rd Qu.    Max. 
##   7.200   7.400   7.550   7.600   7.825   8.100
```

```
## 
##  Shapiro-Wilk normality test
## 
## data:  pre_inf_metrics$red_length
## W = 0.92686, p-value = 0.4178
```

```
##    Min. 1st Qu.  Median    Mean 3rd Qu.    Max. 
##  0.8883  0.9480  1.0152  1.0000  1.0459  1.0918
```

```
## 
##  Shapiro-Wilk normality test
## 
## data:  pre_inf_metrics$red_cond
## W = 0.94698, p-value = 0.6329
```

```
##    Min. 1st Qu.  Median    Mean 3rd Qu.    Max. 
##   3.750   4.790   4.940   5.375   5.335   9.300
```

```
## 
##  Shapiro-Wilk normality test
## 
## data:  pre_inf_metrics$yellow_weight
## W = 0.71842, p-value = 0.001474
```

```
##    Min. 1st Qu.  Median    Mean 3rd Qu.    Max. 
##    6.60    7.25    7.45    7.53    7.60    9.00
```

```
## 
##  Shapiro-Wilk normality test
## 
## data:  pre_inf_metrics$yellow_length
## W = 0.87915, p-value = 0.1276
```

```
##    Min. 1st Qu.  Median    Mean 3rd Qu.    Max. 
##   0.926   1.010   1.027   1.030   1.081   1.089
```

```
## 
##  Shapiro-Wilk normality test
## 
## data:  pre_inf_metrics$yellow_cond
## W = 0.91686, p-value = 0.3315
```

```
##    Min. 1st Qu.  Median    Mean 3rd Qu.    Max. 
##   15.12   18.20   20.39   20.39   22.11   27.17
```

```
## 
##  Shapiro-Wilk normality test
## 
## data:  post_inf_metrics$blue_weight
## W = 0.97804, p-value = 0.9538
```

```
##    Min. 1st Qu.  Median    Mean 3rd Qu.    Max.    NA's 
##   10.00   10.43   11.10   10.84   11.13   11.50       2
```

```
## 
##  Shapiro-Wilk normality test
## 
## data:  post_inf_metrics$blue_length
## W = 0.88359, p-value = 0.2037
```

```
##    Min. 1st Qu.  Median    Mean 3rd Qu.    Max. 
##  0.0000  0.9878  1.0284  0.8373  1.0643  1.1170
```

```
## 
##  Shapiro-Wilk normality test
## 
## data:  post_inf_metrics$blue_cond
## W = 0.59858, p-value = 5.32e-05
```

```
##    Min. 1st Qu.  Median    Mean 3rd Qu.    Max. 
##   12.28   17.77   19.80   19.23   21.08   25.30
```

```
## 
##  Shapiro-Wilk normality test
## 
## data:  post_inf_metrics$orange_weight
## W = 0.96705, p-value = 0.8622
```

```
##    Min. 1st Qu.  Median    Mean 3rd Qu.    Max. 
##    9.60   10.72   10.85   10.80   11.07   11.60
```

```
## 
##  Shapiro-Wilk normality test
## 
## data:  post_inf_metrics$orange_length
## W = 0.91923, p-value = 0.3506
```

```
##    Min. 1st Qu.  Median    Mean 3rd Qu.    Max. 
##  0.9479  0.9886  1.0180  1.0504  1.1142  1.1930
```

```
## 
##  Shapiro-Wilk normality test
## 
## data:  post_inf_metrics$orange_cond
## W = 0.90284, p-value = 0.2353
```

```
##    Min. 1st Qu.  Median    Mean 3rd Qu.    Max. 
##   12.50   16.36   17.16   20.02   23.35   35.09
```

```
## 
##  Shapiro-Wilk normality test
## 
## data:  post_inf_metrics$green_weight
## W = 0.88355, p-value = 0.1433
```

```
##    Min. 1st Qu.  Median    Mean 3rd Qu.    Max. 
##    9.50   10.45   10.85   11.12   11.68   13.10
```

```
## 
##  Shapiro-Wilk normality test
## 
## data:  post_inf_metrics$green_length
## W = 0.95867, p-value = 0.7706
```

```
##    Min. 1st Qu.  Median    Mean 3rd Qu.    Max. 
##  0.8223  0.9213  1.0114  0.9824  1.0396  1.0850
```

```
## 
##  Shapiro-Wilk normality test
## 
## data:  post_inf_metrics$green_cond
## W = 0.92494, p-value = 0.4
```

```
##    Min. 1st Qu.  Median    Mean 3rd Qu.    Max. 
##   12.81   18.91   20.51   20.30   22.28   24.33
```

```
## 
##  Shapiro-Wilk normality test
## 
## data:  post_inf_metrics$red_weight
## W = 0.91506, p-value = 0.3176
```

```
##    Min. 1st Qu.  Median    Mean 3rd Qu.    Max. 
##    9.90   11.03   11.30   11.18   11.45   11.90
```

```
## 
##  Shapiro-Wilk normality test
## 
## data:  post_inf_metrics$red_length
## W = 0.87766, p-value = 0.1227
```

```
##    Min. 1st Qu.  Median    Mean 3rd Qu.    Max. 
##  0.9177  0.9752  0.9882  1.0000  0.9987  1.1120
```

```
## 
##  Shapiro-Wilk normality test
## 
## data:  post_inf_metrics$red_cond
## W = 0.86794, p-value = 0.09459
```

```
##    Min. 1st Qu.  Median    Mean 3rd Qu.    Max. 
##   14.28   16.26   17.73   18.32   20.61   22.76
```

```
## 
##  Shapiro-Wilk normality test
## 
## data:  post_inf_metrics$yellow_weight
## W = 0.92784, p-value = 0.427
```

```
##    Min. 1st Qu.  Median    Mean 3rd Qu.    Max. 
##   10.20   10.43   10.80   10.87   11.05   12.00
```

```
## 
##  Shapiro-Wilk normality test
## 
## data:  post_inf_metrics$yellow_length
## W = 0.90612, p-value = 0.2554
```

```
##    Min. 1st Qu.  Median    Mean 3rd Qu.    Max. 
##  0.9127  0.9323  0.9891  0.9846  1.0247  1.0749
```

```
## 
##  Shapiro-Wilk normality test
## 
## data:  post_inf_metrics$yellow_cond
## W = 0.92241, p-value = 0.3775
```

As by far the majority of the datasets are identified as belonging to a Gaussian distribution according to the Shapiro-Wilk tests, and as visual inspection of the QQ-plots suggests that Shaprio-Wilk P-values > 0.05 are most likely due to single outliers, it is assumed that these data follow an underlying Gaussian distribution.

Testing for significant differences at each time point:

```
## Day 34 - Length
```

```
##             Df Sum Sq Mean Sq F value Pr(>F)
## tm           4  0.217  0.0543   0.258  0.903
## Residuals   45  9.482  0.2107
```

```
## Day 0 - Length
```

```
##             Df Sum Sq Mean Sq F value Pr(>F)
## tm           4  0.221  0.0552    0.36  0.836
## Residuals   45  6.909  0.1535
```

```
## Day 0 - Condition factor
```

```
##             Df Sum Sq  Mean Sq F value Pr(>F)
## tm           4 0.0307 0.007663   1.058  0.388
## Residuals   45 0.3260 0.007244
```

```
## Day 34 - Weight
```

```
##             Df Sum Sq Mean Sq F value Pr(>F)
## tm           4   0.43  0.1082   0.129  0.971
## Residuals   45  37.81  0.8402
```

```
## Day 34 - Length
```

```
##             Df Sum Sq Mean Sq F value Pr(>F)
## tm           4  0.217  0.0543   0.258  0.903
## Residuals   45  9.482  0.2107
```

```
## Day 34 - Condition factor
```

```
##             Df Sum Sq  Mean Sq F value Pr(>F)
## tm           4 0.0229 0.005724   1.852  0.136
## Residuals   45 0.1391 0.003091
```

```
## Day 63 - Weight
```

```
##             Df Sum Sq Mean Sq F value Pr(>F)
## tm           4   30.7   7.672   0.417  0.796
## Residuals   45  828.3  18.407
```

```
## Day 63 - Length
```

```
##             Df Sum Sq Mean Sq F value Pr(>F)
## tm           4  1.192  0.2981    0.61  0.657
## Residuals   43 20.997  0.4883               
## 2 observations deleted due to missingness
```

```
## Day 63 - Condition factor
```

```
##             Df  Sum Sq  Mean Sq F value Pr(>F)  
## tm           4 0.04466 0.011164   2.392 0.0654 .
## Residuals   43 0.20074 0.004668                 
## ---
## Signif. codes:  0 '***' 0.001 '**' 0.01 '*' 0.05 '.' 0.1 ' ' 1
## 2 observations deleted due to missingness
```

No statistically significant differences were found.

Plotting figures:

## Fold height - Distal intestine:

Characterization and comparison of intestinal fold height.

Initial characterization of groupwise fold heights: > - Data summary > - Observations (n) > - Histogram of group fold heights > - Test for underlying Gaussian distribution (Q-Q plot + Shapiro test)

```
## PECF - Pre-feeding - summary
```

```
##    Min. 1st Qu.  Median    Mean 3rd Qu.    Max. 
##    76.6   187.6   268.9   278.8   361.3   605.3
```

```
## # A tibble: 1 x 1
##       n
##   <int>
## 1    50
```

```
## 
##  Shapiro-Wilk normality test
## 
## data:  height_early_group1$lenght
## W = 0.97835, p-value = 0.4845
```

```
## BAYP - Pre-feeding - summary
```

```
##    Min. 1st Qu.  Median    Mean 3rd Qu.    Max. 
##    80.9   155.2   180.0   201.3   237.2   374.9
```

```
## # A tibble: 1 x 1
##       n
##   <int>
## 1    51
```

```
## 
##  Shapiro-Wilk normality test
## 
## data:  height_early_group3$lenght
## W = 0.93419, p-value = 0.007232
```

```
## BAYP - Post-infection - summary
```

```
##    Min. 1st Qu.  Median    Mean 3rd Qu.    Max. 
##    73.1   154.3   239.8   267.0   364.4   664.6
```

```
## # A tibble: 1 x 1
##       n
##   <int>
## 1    62
```

```
## 
##  Shapiro-Wilk normality test
## 
## data:  height_late_group1$lenght
## W = 0.9449, p-value = 0.007641
```

```
## BAYP - Post-infection - summary
```

```
##    Min. 1st Qu.  Median    Mean 3rd Qu.    Max. 
##     9.5   186.1   316.2   327.9   439.9   875.5
```

```
## # A tibble: 1 x 1
##       n
##   <int>
## 1    83
```

```
## 
##  Shapiro-Wilk normality test
## 
## data:  height_late_group3$lenght
## W = 0.95932, p-value = 0.01015
```

The majority of the Shapiro-Wilk normality tests indicate that the fold height data do not come from an underlying Gaussian distribution.

# Statistical analysis of fold heights:

Given that the data is not expected to be from an underlying Gaussian distribution, group data are compared for each time point using an unpaired Mann-Whitney U test:

```
## 
##  Wilcoxon rank sum test with continuity correction
## 
## data:  height_early_group1$lenght and height_early_group3$lenght
## W = 1800, p-value = 0.0003672
## alternative hypothesis: true location shift is not equal to 0
```

```
## 
##  Wilcoxon rank sum test with continuity correction
## 
## data:  height_late_group1$lenght and height_late_group3$lenght
## W = 2113.5, p-value = 0.0666
## alternative hypothesis: true location shift is not equal to 0
```

A statistically significant difference in fold heights was found prior to experimental feeding, while no difference was found at the post-infection sampling time point.

## Fold height - Mid intestine:

Characterization and comparison of intestinal fold height.

Initial characterization of groupwise fold heights:  
- Data summary  
- Observations (n)  
- Histogram of group fold heights  
- Test for underlying Gaussian distribution (Q-Q plot + Shapiro test)

```
## PECF - Day 0 - summary
```

```
##    Min. 1st Qu.  Median    Mean 3rd Qu.    Max. 
##    84.5   156.1   189.1   196.9   237.4   331.5
```

```
## # A tibble: 1 x 1
##       n
##   <int>
## 1    71
```

```
## 
##  Shapiro-Wilk normality test
## 
## data:  height_early_mid_group1$height
## W = 0.97924, p-value = 0.2883
```

```
## BAYP - Day 0 - summary
```

```
##    Min. 1st Qu.  Median    Mean 3rd Qu.    Max. 
##    50.6   107.7   163.4   165.8   202.6   328.3
```

```
## # A tibble: 1 x 1
##       n
##   <int>
## 1    67
```

```
## 
##  Shapiro-Wilk normality test
## 
## data:  height_early_mid_group3$height
## W = 0.95768, p-value = 0.02276
```

```
## PECF - Day 63 - summary
```

```
##    Min. 1st Qu.  Median    Mean 3rd Qu.    Max. 
##    70.4   142.9   243.6   273.1   416.4   580.6
```

```
## # A tibble: 1 x 1
##       n
##   <int>
## 1    82
```

```
## 
##  Shapiro-Wilk normality test
## 
## data:  height_late_mid_group1$height
## W = 0.91881, p-value = 6.842e-05
```

```
## BAYP - Day 63 - summary
```

```
##    Min. 1st Qu.  Median    Mean 3rd Qu.    Max. 
##    43.5   191.2   286.7   305.9   420.8   667.4
```

```
## # A tibble: 1 x 1
##       n
##   <int>
## 1   175
```

```
## 
##  Shapiro-Wilk normality test
## 
## data:  height_late_mid_group3$height
## W = 0.97251, p-value = 0.001536
```

# Statistical analysis of fold heights:

As the results from the Shapiro-Wilk tests, as well as the visual inspections imply that the measured fold heights are not expected to be from an underlying Gaussian distribution, group data are compared for each time point using an unpaired Mann-Whitney U test:

```
## 
##  Wilcoxon rank sum test with continuity correction
## 
## data:  height_early_mid_group1$height and height_early_mid_group3$height
## W = 3056, p-value = 0.003926
## alternative hypothesis: true location shift is not equal to 0
```

```
## 
##  Wilcoxon rank sum test with continuity correction
## 
## data:  height_late_mid_group1$height and height_late_mid_group3$height
## W = 6225, p-value = 0.08737
## alternative hypothesis: true location shift is not equal to 0
```

A statistically significant difference in fold heights was found prior to experimental feeding, while no difference was found at the post-infection sampling time point.

## Visualization:

Comparing and presenting fold heights from both intestinal regions in dot-charts:

# Survival analysis

The results from the experimental infections are analysed by plotting Kaplan-Meier survival curves and applying a log-rank analysis to groups of curves. As infections were performed in quadruplicates for each feed code, the replicate curves within each group were tested for significant differences prior to any pooling of data:

## Individual groupwise comparisons of replicates

### Comparing the PECF replicates:

```
## Call: survfit(formula = surv.blue ~ label, data = Collected_data_for_R_analysis)
## 
##                 label=1 
##  time n.risk n.event survival std.err lower 95% CI upper 95% CI
##     4     65      17    0.738  0.0545        0.639        0.853
##     5     48       9    0.600  0.0608        0.492        0.732
##     6     39       9    0.462  0.0618        0.355        0.600
##     7     30       5    0.385  0.0603        0.283        0.523
##     8     25       3    0.338  0.0587        0.241        0.475
##     9     22       3    0.292  0.0564        0.200        0.427
##    13     19       1    0.277  0.0555        0.187        0.410
##    14     18       1    0.262  0.0545        0.174        0.393
## 
##                 label=7 
##  time n.risk n.event survival std.err lower 95% CI upper 95% CI
##     1     65       1    0.985  0.0153        0.955        1.000
##     3     64       1    0.969  0.0214        0.928        1.000
##     4     63       9    0.831  0.0465        0.744        0.927
##     5     54      17    0.569  0.0614        0.461        0.703
##     6     37      11    0.400  0.0608        0.297        0.539
##     7     26       1    0.385  0.0603        0.283        0.523
##     8     25       5    0.308  0.0572        0.214        0.443
##     9     20       1    0.292  0.0564        0.200        0.427
##    11     19       1    0.277  0.0555        0.187        0.410
##    12     18       1    0.262  0.0545        0.174        0.393
## 
##                 label=13 
##  time n.risk n.event survival std.err lower 95% CI upper 95% CI
##     4     64      13    0.797  0.0503       0.7042        0.902
##     5     51      22    0.453  0.0622       0.3462        0.593
##     6     29      10    0.297  0.0571       0.2036        0.433
##     7     19       6    0.203  0.0503       0.1250        0.330
##     9     13       1    0.188  0.0488       0.1126        0.312
##    11     12       3    0.141  0.0435       0.0767        0.258
## 
##                 label=19 
##  time n.risk n.event survival std.err lower 95% CI upper 95% CI
##     4     65       8    0.877  0.0407        0.801        0.961
##     5     57      25    0.492  0.0620        0.385        0.630
##     6     32       8    0.369  0.0599        0.269        0.507
##     7     24       3    0.323  0.0580        0.227        0.459
##     8     21       4    0.262  0.0545        0.174        0.393
##    11     17       2    0.231  0.0523        0.148        0.360
##    12     15       1    0.215  0.0510        0.135        0.343
```

No significant differences between replicates were found.

### Comparing the PEYP replicates:

```
## Call: survfit(formula = surv.orange ~ label, data = Surv_orange)
## 
##                 label=2 
##  time n.risk n.event survival std.err lower 95% CI upper 95% CI
##     3     64       3    0.953  0.0264        0.903        1.000
##     4     61      20    0.641  0.0600        0.533        0.770
##     5     41      10    0.484  0.0625        0.376        0.624
##     6     31       9    0.344  0.0594        0.245        0.482
##     7     22       1    0.328  0.0587        0.231        0.466
##     8     21       3    0.281  0.0562        0.190        0.416
##     9     18       2    0.250  0.0541        0.164        0.382
##    10     16       1    0.234  0.0530        0.151        0.365
##    12     15       1    0.219  0.0517        0.138        0.348
## 
##                 label=8 
##  time n.risk n.event survival std.err lower 95% CI upper 95% CI
##     3     68       2    0.971  0.0205        0.931        1.000
##     4     66      16    0.735  0.0535        0.638        0.848
##     5     50      15    0.515  0.0606        0.409        0.648
##     6     35       5    0.441  0.0602        0.338        0.576
##     7     30       3    0.397  0.0593        0.296        0.532
##     8     27       4    0.338  0.0574        0.243        0.472
##     9     23       3    0.294  0.0553        0.204        0.425
##    10     20       1    0.279  0.0544        0.191        0.409
##    13     19       1    0.265  0.0535        0.178        0.393
## 
##                 label=14 
##  time n.risk n.event survival std.err lower 95% CI upper 95% CI
##     4     65      15    0.769  0.0523        0.673        0.879
##     5     50      13    0.569  0.0614        0.461        0.703
##     6     37      13    0.369  0.0599        0.269        0.507
##     7     24       4    0.308  0.0572        0.214        0.443
##     8     20       3    0.262  0.0545        0.174        0.393
##    12     17       3    0.215  0.0510        0.135        0.343
##    14     14       1    0.200  0.0496        0.123        0.325
## 
##                 label=20 
##  time n.risk n.event survival std.err lower 95% CI upper 95% CI
##     3     65       2    0.969  0.0214        0.928        1.000
##     4     63       9    0.831  0.0465        0.744        0.927
##     5     54      13    0.631  0.0599        0.524        0.760
##     6     41      13    0.431  0.0614        0.326        0.570
##     7     28       4    0.369  0.0599        0.269        0.507
##     8     24       3    0.323  0.0580        0.227        0.459
##     9     21       5    0.246  0.0534        0.161        0.377
##    11     16       2    0.215  0.0510        0.135        0.343
```

No significant differences were found between replicates.

### Comparing the BAYP replicates:

```
## Call: survfit(formula = Surv.green ~ label, data = surv.green)
## 
##                 label=3 
##  time n.risk n.event survival std.err lower 95% CI upper 95% CI
##     3     65       1    0.985  0.0153        0.955        1.000
##     4     64      26    0.585  0.0611        0.476        0.718
##     5     38      12    0.400  0.0608        0.297        0.539
##     6     26       3    0.354  0.0593        0.255        0.491
##     7     23       4    0.292  0.0564        0.200        0.427
##     9     19       3    0.246  0.0534        0.161        0.377
##    10     16       3    0.200  0.0496        0.123        0.325
##    12     13       1    0.185  0.0481        0.111        0.308
## 
##                 label=9 
##  time n.risk n.event survival std.err lower 95% CI upper 95% CI
##     3     67       1    0.985  0.0148        0.956        1.000
##     4     66      21    0.672  0.0574        0.568        0.794
##     5     45      12    0.493  0.0611        0.386        0.628
##     6     33       7    0.388  0.0595        0.287        0.524
##     7     26       2    0.358  0.0586        0.260        0.494
##     8     24       2    0.328  0.0574        0.233        0.462
##     9     22       1    0.313  0.0567        0.220        0.447
##    11     21       1    0.299  0.0559        0.207        0.431
## 
##                 label=15 
##  time n.risk n.event survival std.err lower 95% CI upper 95% CI
##     3     65       2    0.969  0.0214       0.9281        1.000
##     4     63      25    0.585  0.0611       0.4763        0.718
##     5     38      13    0.385  0.0603       0.2828        0.523
##     6     25       9    0.246  0.0534       0.1609        0.377
##     7     16       4    0.185  0.0481       0.1108        0.308
##     9     12       1    0.169  0.0465       0.0988        0.290
##    12     11       1    0.154  0.0448       0.0870        0.272
##    14     10       1    0.138  0.0428       0.0755        0.254
## 
##                 label=21 
##  time n.risk n.event survival std.err lower 95% CI upper 95% CI
##     1     65       3    0.954  0.0260       0.9042        1.000
##     3     62       1    0.938  0.0298       0.8818        0.999
##     4     61       6    0.846  0.0448       0.7628        0.939
##     5     55      23    0.492  0.0620       0.3846        0.630
##     6     32      15    0.262  0.0545       0.1738        0.393
##     7     17       2    0.231  0.0523       0.1481        0.360
##     8     15       2    0.200  0.0496       0.1230        0.325
##     9     13       2    0.169  0.0465       0.0988        0.290
##    10     11       1    0.154  0.0448       0.0870        0.272
##    11     10       1    0.138  0.0428       0.0755        0.254
```

No significant differences were found between replicates.

### Comparing the Control replicates:

```
## Call: survfit(formula = Surv.red ~ label, data = Surv_red)
## 
##                 label=4 
##  time n.risk n.event survival std.err lower 95% CI upper 95% CI
##     3     65       1    0.985  0.0153        0.955        1.000
##     4     64      21    0.662  0.0587        0.556        0.787
##     5     43      14    0.446  0.0617        0.340        0.585
##     6     29       6    0.354  0.0593        0.255        0.491
##     7     23       5    0.277  0.0555        0.187        0.410
##     8     18       1    0.262  0.0545        0.174        0.393
##     9     17       1    0.246  0.0534        0.161        0.377
##    14     16       1    0.231  0.0523        0.148        0.360
## 
##                 label=10 
##  time n.risk n.event survival std.err lower 95% CI upper 95% CI
##     4     65      20    0.692  0.0572       0.5887        0.814
##     5     45      10    0.538  0.0618       0.4299        0.674
##     6     35      11    0.369  0.0599       0.2687        0.507
##     7     24       8    0.246  0.0534       0.1609        0.377
##     8     16       5    0.169  0.0465       0.0988        0.290
##     9     11       2    0.138  0.0428       0.0755        0.254
##    12      9       1    0.123  0.0407       0.0643        0.236
##    14      8       1    0.108  0.0384       0.0535        0.217
## 
##                 label=16 
##  time n.risk n.event survival std.err lower 95% CI upper 95% CI
##     1     65       1    0.985  0.0153        0.955        1.000
##     3     64       3    0.938  0.0298        0.882        0.999
##     4     61      19    0.646  0.0593        0.540        0.774
##     5     42      15    0.415  0.0611        0.311        0.554
##     6     27       9    0.277  0.0555        0.187        0.410
##     7     18       3    0.231  0.0523        0.148        0.360
##     8     15       1    0.215  0.0510        0.135        0.343
##     9     14       1    0.200  0.0496        0.123        0.325
##    13     13       1    0.185  0.0481        0.111        0.308
## 
##                 label=22 
##  time n.risk n.event survival std.err lower 95% CI upper 95% CI
##     3     64       3    0.953  0.0264        0.903        1.000
##     4     61      11    0.781  0.0517        0.686        0.889
##     5     50      15    0.547  0.0622        0.438        0.684
##     6     35       8    0.422  0.0617        0.317        0.562
##     7     27       3    0.375  0.0605        0.273        0.515
##     8     24       3    0.328  0.0587        0.231        0.466
##    10     21       1    0.312  0.0579        0.217        0.449
##    11     20       2    0.281  0.0562        0.190        0.416
##    12     18       2    0.250  0.0541        0.164        0.382
```

No significant differences were found.

### Comparing the PEBP replicates:

```
## Call: survfit(formula = Surv.yellow ~ label, data = Surv_yellow)
## 
##                 label=5 
##  time n.risk n.event survival std.err lower 95% CI upper 95% CI
##     3     65       3    0.954  0.0260        0.904        1.000
##     4     62      10    0.800  0.0496        0.708        0.903
##     5     52      15    0.569  0.0614        0.461        0.703
##     6     37      12    0.385  0.0603        0.283        0.523
##     7     25       5    0.308  0.0572        0.214        0.443
##     9     20       3    0.262  0.0545        0.174        0.393
##    10     17       1    0.246  0.0534        0.161        0.377
##    11     16       1    0.231  0.0523        0.148        0.360
##    12     15       1    0.215  0.0510        0.135        0.343
##    13     14       1    0.200  0.0496        0.123        0.325
##    19     13       1    0.185  0.0481        0.111        0.308
## 
##                 label=11 
##  time n.risk n.event survival std.err lower 95% CI upper 95% CI
##     3     67       4    0.940  0.0289        0.885        0.999
##     4     63      11    0.776  0.0509        0.682        0.883
##     5     52      15    0.552  0.0608        0.445        0.685
##     6     37       8    0.433  0.0605        0.329        0.569
##     7     29       8    0.313  0.0567        0.220        0.447
##     8     21       1    0.299  0.0559        0.207        0.431
##     9     20       3    0.254  0.0532        0.168        0.383
##    11     17       1    0.239  0.0521        0.156        0.366
## 
##                 label=17 
##  time n.risk n.event survival std.err lower 95% CI upper 95% CI
##     3     65       1    0.985  0.0153        0.955        1.000
##     4     64      23    0.631  0.0599        0.524        0.760
##     5     41      12    0.446  0.0617        0.340        0.585
##     6     29       4    0.385  0.0603        0.283        0.523
##     7     25       5    0.308  0.0572        0.214        0.443
##     8     20       1    0.292  0.0564        0.200        0.427
##    10     19       2    0.262  0.0545        0.174        0.393
##    12     17       2    0.231  0.0523        0.148        0.360
## 
##                 label=23 
##  time n.risk n.event survival std.err lower 95% CI upper 95% CI
##     3     65       3    0.954  0.0260       0.9042        1.000
##     4     62      11    0.785  0.0510       0.6908        0.891
##     5     51      11    0.615  0.0603       0.5078        0.746
##     6     40       9    0.477  0.0620       0.3697        0.615
##     7     31       7    0.369  0.0599       0.2687        0.507
##     8     24       8    0.246  0.0534       0.1609        0.377
##     9     16       2    0.215  0.0510       0.1354        0.343
##    10     14       2    0.185  0.0481       0.1108        0.308
##    11     12       1    0.169  0.0465       0.0988        0.290
##    12     11       1    0.154  0.0448       0.0870        0.272
```

No significant differences were found.

## Testing for differences between pooled groups

As no significant differences between replicates were found, data from all replicates in each group were pooled. Now the data from each group can be compared:

```
## Call: survfit(formula = Surv.pooled ~ label, data = Surv_pooled)
## 
##                 label=Control 
##  time n.risk n.event survival std.err lower 95% CI upper 95% CI
##     1    259       1    0.996 0.00385        0.989        1.000
##     3    258       7    0.969 0.01075        0.948        0.990
##     4    251      71    0.695 0.02861        0.641        0.753
##     5    180      54    0.486 0.03106        0.429        0.551
##     6    126      34    0.355 0.02974        0.301        0.419
##     7     92      19    0.282 0.02796        0.232        0.342
##     8     73      10    0.243 0.02666        0.196        0.302
##     9     63       4    0.228 0.02606        0.182        0.285
##    10     59       1    0.224 0.02590        0.179        0.281
##    11     58       2    0.216 0.02558        0.171        0.273
##    12     56       3    0.205 0.02507        0.161        0.260
##    13     53       1    0.201 0.02489        0.157        0.256
##    14     52       2    0.193 0.02452        0.150        0.248
## 
##                 label=PECF 
##  time n.risk n.event survival std.err lower 95% CI upper 95% CI
##     1    259       1    0.996 0.00385        0.989        1.000
##     3    258       1    0.992 0.00544        0.982        1.000
##     4    257      47    0.811 0.02434        0.764        0.860
##     5    210      73    0.529 0.03102        0.472        0.593
##     6    137      38    0.382 0.03019        0.327        0.446
##     7     99      15    0.324 0.02909        0.272        0.387
##     8     84      12    0.278 0.02784        0.228        0.338
##     9     72       5    0.259 0.02721        0.210        0.318
##    11     67       6    0.236 0.02637        0.189        0.293
##    12     61       2    0.228 0.02606        0.182        0.285
##    13     59       1    0.224 0.02590        0.179        0.281
##    14     58       1    0.220 0.02574        0.175        0.277
## 
##                 label=PEBP 
##  time n.risk n.event survival std.err lower 95% CI upper 95% CI
##     3    262      11    0.958  0.0124        0.934        0.983
##     4    251      55    0.748  0.0268        0.697        0.803
##     5    196      53    0.546  0.0308        0.489        0.610
##     6    143      33    0.420  0.0305        0.364        0.484
##     7    110      25    0.324  0.0289        0.272        0.386
##     8     85      10    0.286  0.0279        0.236        0.347
##     9     75       8    0.256  0.0270        0.208        0.314
##    10     67       5    0.237  0.0263        0.190        0.294
##    11     62       3    0.225  0.0258        0.180        0.282
##    12     59       4    0.210  0.0252        0.166        0.266
##    13     55       1    0.206  0.0250        0.163        0.261
##    19     54       1    0.202  0.0248        0.159        0.257
## 
##                 label=PEYP 
##  time n.risk n.event survival std.err lower 95% CI upper 95% CI
##     3    262       7    0.973 0.00996        0.954        0.993
##     4    255      60    0.744 0.02695        0.693        0.799
##     5    195      51    0.550 0.03074        0.493        0.613
##     6    144      40    0.397 0.03023        0.342        0.461
##     7    104      12    0.351 0.02949        0.298        0.414
##     8     92      13    0.302 0.02835        0.251        0.363
##     9     79      10    0.263 0.02721        0.215        0.322
##    10     69       2    0.256 0.02695        0.208        0.314
##    11     67       2    0.248 0.02668        0.201        0.306
##    12     65       4    0.233 0.02611        0.187        0.290
##    13     61       1    0.229 0.02596        0.183        0.286
##    14     60       1    0.225 0.02581        0.180        0.282
## 
##                 label=BAYP 
##  time n.risk n.event survival std.err lower 95% CI upper 95% CI
##     1    262       3    0.989 0.00657        0.976        1.000
##     3    259       5    0.969 0.01063        0.949        0.991
##     4    254      78    0.672 0.02901        0.617        0.731
##     5    176      60    0.443 0.03069        0.387        0.507
##     6    116      34    0.313 0.02865        0.262        0.374
##     7     82      12    0.267 0.02734        0.219        0.327
##     8     70       4    0.252 0.02682        0.204        0.310
##     9     66       7    0.225 0.02581        0.180        0.282
##    10     59       4    0.210 0.02516        0.166        0.266
##    11     55       2    0.202 0.02482        0.159        0.257
##    12     53       2    0.195 0.02446        0.152        0.249
##    14     51       1    0.191 0.02428        0.149        0.245
```

No significant differences are found between any groups.

Finally, the hazard ratio analysis is performed with Control feed group as reference using Cox Proportional Hazards analysis:

No significant differences are seen

## Citations:

```
## 
## To cite R in publications use:
## 
##   R Core Team (2018). R: A language and environment for statistical
##   computing. R Foundation for Statistical Computing, Vienna, Austria.
##   URL https://www.R-project.org/.
## 
## A BibTeX entry for LaTeX users is
## 
##   @Manual{,
##     title = {R: A Language and Environment for Statistical Computing},
##     author = {{R Core Team}},
##     organization = {R Foundation for Statistical Computing},
##     address = {Vienna, Austria},
##     year = {2018},
##     url = {https://www.R-project.org/},
##   }
## 
## We have invested a lot of time and effort in creating R, please cite it
## when using it for data analysis. See also 'citation("pkgname")' for
## citing R packages.
```

```
## 
## To cite ggplot2 in publications, please use:
## 
##   H. Wickham. ggplot2: Elegant Graphics for Data Analysis.
##   Springer-Verlag New York, 2016.
## 
## A BibTeX entry for LaTeX users is
## 
##   @Book{,
##     author = {Hadley Wickham},
##     title = {ggplot2: Elegant Graphics for Data Analysis},
##     publisher = {Springer-Verlag New York},
##     year = {2016},
##     isbn = {978-3-319-24277-4},
##     url = {https://ggplot2.tidyverse.org},
##   }
```

```
## 
## Therneau T (2020). _A Package for Survival Analysis in R_. R package
## version 3.1-11, <URL: https://CRAN.R-project.org/package=survival>.
## 
## Terry M. Therneau, Patricia M. Grambsch (2000). _Modeling Survival
## Data: Extending the Cox Model_. Springer, New York. ISBN 0-387-98784-3.
## 
## To see these entries in BibTeX format, use 'print(<citation>,
## bibtex=TRUE)', 'toBibtex(.)', or set
## 'options(citation.bibtex.max=999)'.
```

```
## 
## To cite package 'survminer' in publications use:
## 
##   Alboukadel Kassambara, Marcin Kosinski and Przemyslaw Biecek (2019).
##   survminer: Drawing Survival Curves using 'ggplot2'. R package version
##   0.4.6. https://CRAN.R-project.org/package=survminer
## 
## A BibTeX entry for LaTeX users is
## 
##   @Manual{,
##     title = {survminer: Drawing Survival Curves using 'ggplot2'},
##     author = {Alboukadel Kassambara and Marcin Kosinski and Przemyslaw Biecek},
##     year = {2019},
##     note = {R package version 0.4.6},
##     url = {https://CRAN.R-project.org/package=survminer},
##   }
```

```
## 
## To cite package 'readxl' in publications use:
## 
##   Hadley Wickham and Jennifer Bryan (2019). readxl: Read Excel Files. R
##   package version 1.3.1. https://CRAN.R-project.org/package=readxl
## 
## A BibTeX entry for LaTeX users is
## 
##   @Manual{,
##     title = {readxl: Read Excel Files},
##     author = {Hadley Wickham and Jennifer Bryan},
##     year = {2019},
##     note = {R package version 1.3.1},
##     url = {https://CRAN.R-project.org/package=readxl},
##   }
```

```
## 
## To cite package 'ggpubr' in publications use:
## 
##   Alboukadel Kassambara (2020). ggpubr: 'ggplot2' Based Publication
##   Ready Plots. R package version 0.2.5.
##   https://CRAN.R-project.org/package=ggpubr
## 
## A BibTeX entry for LaTeX users is
## 
##   @Manual{,
##     title = {ggpubr: 'ggplot2' Based Publication Ready Plots},
##     author = {Alboukadel Kassambara},
##     year = {2020},
##     note = {R package version 0.2.5},
##     url = {https://CRAN.R-project.org/package=ggpubr},
##   }
```

```
## 
## To cite package 'dplyr' in publications use:
## 
##   Hadley Wickham, Romain François, Lionel Henry and Kirill Müller
##   (2020). dplyr: A Grammar of Data Manipulation. R package version
##   0.8.5. https://CRAN.R-project.org/package=dplyr
## 
## A BibTeX entry for LaTeX users is
## 
##   @Manual{,
##     title = {dplyr: A Grammar of Data Manipulation},
##     author = {Hadley Wickham and Romain François and Lionel Henry and Kirill Müller},
##     year = {2020},
##     note = {R package version 0.8.5},
##     url = {https://CRAN.R-project.org/package=dplyr},
##   }
```

```
## 
## To cite package 'RColorBrewer' in publications use:
## 
##   Erich Neuwirth (2014). RColorBrewer: ColorBrewer Palettes. R package
##   version 1.1-2. https://CRAN.R-project.org/package=RColorBrewer
## 
## A BibTeX entry for LaTeX users is
## 
##   @Manual{,
##     title = {RColorBrewer: ColorBrewer Palettes},
##     author = {Erich Neuwirth},
##     year = {2014},
##     note = {R package version 1.1-2},
##     url = {https://CRAN.R-project.org/package=RColorBrewer},
##   }
```

```
## 
## To cite package 'ggradar' in publications use:
## 
##   Ricardo Bion (2019). ggradar: Create radar charts using ggplot2. R
##   package version 0.2.
## 
## A BibTeX entry for LaTeX users is
## 
##   @Manual{,
##     title = {ggradar: Create radar charts using ggplot2},
##     author = {Ricardo Bion},
##     year = {2019},
##     note = {R package version 0.2},
##   }
```

```
## 
## To cite package 'scales' in publications use:
## 
##   Hadley Wickham and Dana Seidel (2019). scales: Scale Functions for
##   Visualization. R package version 1.1.0.
##   https://CRAN.R-project.org/package=scales
## 
## A BibTeX entry for LaTeX users is
## 
##   @Manual{,
##     title = {scales: Scale Functions for Visualization},
##     author = {Hadley Wickham and Dana Seidel},
##     year = {2019},
##     note = {R package version 1.1.0},
##     url = {https://CRAN.R-project.org/package=scales},
##   }
```

```
## 
## To cite package 'tibble' in publications use:
## 
##   Kirill Müller and Hadley Wickham (2020). tibble: Simple Data Frames.
##   R package version 3.0.0. https://CRAN.R-project.org/package=tibble
## 
## A BibTeX entry for LaTeX users is
## 
##   @Manual{,
##     title = {tibble: Simple Data Frames},
##     author = {Kirill Müller and Hadley Wickham},
##     year = {2020},
##     note = {R package version 3.0.0},
##     url = {https://CRAN.R-project.org/package=tibble},
##   }
```

```
## 
## To cite package 'fmsb' in publications use:
## 
##   Minato Nakazawa (2019). fmsb: Functions for Medical Statistics Book
##   with some Demographic Data. R package version 0.7.0.
##   https://CRAN.R-project.org/package=fmsb
## 
## A BibTeX entry for LaTeX users is
## 
##   @Manual{,
##     title = {fmsb: Functions for Medical Statistics Book with some Demographic Data},
##     author = {Minato Nakazawa},
##     year = {2019},
##     note = {R package version 0.7.0},
##     url = {https://CRAN.R-project.org/package=fmsb},
##   }
## 
## ATTENTION: This citation information has been auto-generated from the
## package DESCRIPTION file and may need manual editing, see
## 'help("citation")'.
```
